# Methods for tagging whale sharks: insights into performance and best practices with a focus on clamp attachments

**DOI:** 10.1186/s40317-026-00462-4

**Published:** 2026-05-05

**Authors:** Freya C. Womersley, Sofia Green, Alberto Garcia-Baciero, Ronan Conlon, Amy L. Jeffries, Matt J. Waller, Sara S. Ratão, Nuno Queiroz, Pedro Afonso, Gonzalo Araujo, Adam Barnett, Christine Barry, Michael L. Berumen, Farukhkha Bloch, Ginevra Boldrocchi, Camrin D. Braun, Ryan Caillouet, Constance Chapman, Jesse E. M. Cochran, Rafael de la Parra, Stella Diamant, Alistair D. M. Dove, Matthew Dunbabin, Mark V. Erdmann, Luciana C. Ferreira, Richard Fitzpatrick, Jorge Fontes, Adrian C. Gleiss, Jonathan R. Green, Lucas P. Griffin, Curtice R. Griffin, Royale S. Hardenstine, Abdi Hassan, Alex R. Hearn, Jill M. Hendon, Mochamad Iqbal Herwata Putra, Eric Hoffmayer, Lisa Hoopes, Robert E. Hueter, Sajan John, Jake Levenson, Sonny Lewis, Bruno C. L. Macena, Mark G. Meekan, Ingo B. Miller, Brad Norman, Jens Paulsen, Cameron Perry, Simon J. Pierce, Samantha D. Reynolds, David P. Robinson, Christoph A. Rohner, Jennifer Schmidt, Edy Setyawan, Abraham B. Sianipar, Jamison Smith, Simon R. Thorrold, Michele Thums, Rory Wilson, Emily J. Southall, David W. Sims

**Affiliations:** 1 Marine Biological Association, The Laboratory, Citadel Hill, Plymouth, PL1 2PB UK; 2 Galapagos Whale Shark Project, Puerto Ayora, Galapagos Islands Ecuador; 3 Whale Shark Mexico-Conexiones Terramar, Independencia 106, 23000 La Paz, BCS Mexico; 4 Instituto Politécnico Nacional, Centro Interdisciplinario de Ciencias Marinas (CICIMAR). Av. Instituto Politécnico Nacional S/N, 23096 La Paz, BCS Mexico; 5 Ocean and Earth Science, National Oceanography Centre Southampton, University of Southampton, Southampton, UK; 6 Departamento de Biologia, Faculdade de Ciências da Universidade Do Porto, 4099-002 Porto, Portugal; 7 CIBIO, Centro de Investigação Em Biodiversidade E Recursos Genéticos, InBIO Laboratório Associado, Universidade Do Porto, Campus de Vairão, 4485-661 Vairão, Portugal; 8 BIOPOLIS Program in Genomics, Biodiversity and Land Planning, CIBIO, 4485-661 Vairão, Portugal; 9 Institute of Marine Research - IMAR, Department of Oceanography and Fisheries, University of the Azores, 9900-140 Horta, Portugal; 10 Institute of Marine Sciences - OKEANOS, University of the Azores, 9900-140 Horta, Portugal; 11 Marine Research and Conservation Foundation, Lydeard St Lawrence, Somerset, TA4 3SJ UK; 12 Environmental Science Program, Department of Biological and Environmental Sciences, College of Arts and Sciences, Qatar University, Doha, Qatar; 13 Biopixel Oceans Foundation, Cairns, Australia; 14 Centre for Sustainable Aquatic Ecosystems, Harry Butler Institute, Murdoch University, Murdoch, WA Australia; 15 Australian Institute of Marine Science (AIMS), Crawley, WA Australia; 16 Environmental and Conservation Sciences, Murdoch University, Murdoch, WA Australia; 17 Marine Science Program, Division of Biological and Environmental Science and Engineering, King Abdullah University of Science and Technology, Thuwal, Kingdom of Saudi Arabia; 18 Pan India Whale Shark Conservation Project, Wildlife Trust of India, F-13, Sector 8, Noida, NCR 201301 India; 19 University of Insubria, Varese, VA Italy; 20 Biology Department, Woods Hole Oceanographic Institution, Woods Hole, MA 02543 USA; 21 National Marine Fisheries Service, Southeast Fisheries Science Center, Pascagoula, USA; 22 Ch’ooj Ajuail AC, Xel-Ha 28, 77509 Cancún, Quintana Roo Mexico; 23 Madagascar Whale Shark Project, Nosy Be, Madagascar; 24 Museum of Science and History, Jacksonville, USA; 25 Queensland University of Technology, Brisbane, QLD Australia; 26 Conservation International New Zealand, University of Auckland, 23 Symonds Street, Auckland, NZ 1020 New Zealand; 27 Re:Wild, Austin, TX 78767 USA; 28 Department of Integrative Biology, University of South Florida, Tampa, FL 33620 USA; 29 Department of Environmental Conservation, University of Massachusetts Amherst, Amherst, MA 01003 USA; 30 Focal Species Program, Ocean & Science Department, Konservasi Indonesia, Jakarta, Indonesia; 31 Galapagos Science Center, Universidad San Francisco de Quito USFQ, Quito, Ecuador; 32 MigraMar, 2099 Westshore Rd, Bodega Bay, CA 94923 USA; 33 University of Southern Mississippi, Ocean Springs, MS USA; 34 IUCN Center for Species Survival, Georgia Aquarium, Atlanta, GA USA; 35 Mote Marine Laboratory, Sarasota, FL USA; 36 OCEARCH, Park City, UT USA; 37 Bureau of Ocean Energy Management–BOEM, Washington, DC USA; 38 ECOCEAN Inc., Coogee, WA Australia; 39 AIMS@JCU, College of Science and Engineering, James Cook University, Cairns, Australia; 40 Harry Butler Institute, Murdoch University, Murdoch, WA Australia; 41 Marine Megafauna Foundation, West Palm Beach, FL USA; 42 University of the Sunshine Coast, Sippy Downs, QLD Australia; 43 Sundive Research, Byron Bay, NSW 2481 Australia; 44 Shark Research Institute, Princeton, USA; 45 Elasmobranch Institute Indonesia, Denpasar, Bali, 80226 Indonesia; 46 Blue World Research Institute, Cocoa, FL USA; 47 Swansea Lab for Animal Movement, Swansea University, Swansea, Wales; 48 Oceans Institute, University of Western Australia, WA, 6009 Australia

**Keywords:** Animal movement, Spatial ecology, Satellite tracking, Biologging technology, Rhincodon typus, Minimally-invasive techniques, Conservation technology

## Abstract

**Background:**

Biologging and telemetry have transformed our understanding of marine megafauna movement ecology. Yet, methodological constraints continue to limit data quality and deployment duration. Devices recording whale shark (*Rhincodon typus*) behaviours and movements have been used for decades, but they remain challenging to deploy and vary in success. Recently, spring-loaded clamp-based systems have emerged as one of the most widely used approaches to attach electronic tags to the fins of this globally endangered species. Currently, however, no consensus guidelines exist as to how to optimise this approach, potentially leading to continued underperforming deployments limiting analysis potential. Here, we synthesise experiences with clamp-based tagging worldwide through a targeted survey of whale shark researchers. We explore performance and challenges with a view to propose current best practices in the field.

**Results:**

Whale shark researcher responses to the survey highlighted clamp-based systems as a practical and more widely applicable approach than drill-based methods, which are often used to secure tags to other large sharks. They also noted that clamps have greater retention potential and are suitable for a wider range of tags compared to dart-based methods, but are still constrained by design, placement, and deployment conditions. Researchers used a variety of materials and designs to build their own clamps, often facilitated by direct collaboration with each other or key manufacturers. Clamps produced highly variable outcomes, ranging from successful long-term satellite transmissions over 200 days and short-term biologging for 48 h at 20 Hz, to premature detachment and cases of fin damage. For long-term clamps, changes in position on the fin allowed for more stable satellite transmissions over time. Some clamp designs achieved data quantity and quality close to that of drilled deployments, demonstrating their potential to rival traditional methods while offering a less invasive approach. Results emphasised the ongoing need for technological refinement and rigorous evaluation of clamp performance and associated impacts.

**Conclusions:**

Based on collective insights, we present a unified approach to clamp design and positioning, and identify key priorities for advancing this attachment technology, such as aiming for positions b-2 and c-2 on the fin and ensuring the clamp bridge distance (always between 30 and 50 mm) and tension are matched to shark size. Optimising clamp systems could substantially improve our ability to generate high-quality, long-duration movement data while minimising tagging impacts on the animal where possible. This could enhance ecological and conservation research outcomes for endangered whale sharks, with broader implications for tagging other large-bodied marine megafauna.

**Supplementary Information:**

The online version contains supplementary material available at 10.1186/s40317-026-00462-4.

## Background

Amid accelerating global biodiversity loss [1–4], information on the movements, behaviours, and habitat use of animals is becoming recognised as one of the most powerful ways to inform conservation of threatened species [5–7]. Tracking animals with biologging and telemetry devices, for example, can reveal the mechanisms of species loss [8], uncover key areas used by multiple groups [9], and predict distribution shifts in the face of anthropogenic [10] and climate [11] change. These insights are particularly valuable for species that cannot be easily observed directly for long periods and distances, such as large-bodied and highly-mobile marine megafauna. However, actively tracking these animals poses substantial challenges due to their large body size, wide-ranging movements, deep diving with short surfacing times, and their often remote, inaccessible habitats [12].

Tracking technologies have advanced in response to these challenges. Recent improvements in battery life now enable longer tracking durations to capture wide-ranging movements; sensor miniaturisation allows multi-sensor deployments, maximising value of single devices for species that are difficult to tag; advances in data storage and transmission make it possible to store and relay data from animals that surface infrequently; and durability standards have increased, with devices able to withstand sub-zero temperatures and water immersion to depths of up to 2000 m [13]. In parallel, improvements in manufacturing pipelines and access to raw materials have reduced unit costs, making wildlife tracking technologies more accessible than ever, with the market forecast to expand by more than 10% through 2030 [13].

Satellite telemetry and archival biologging specifically – techniques involving externally securing devices to an animal to log their movements, behaviour, physiology and environmental conditions – are now widely used in marine megafauna research [14–16]. However, a fundamental challenge remains: securely and ethically attaching these devices in ways that ensure high-quality data collection and successful data recovery or transmission [17, 18]. Attachment methods such as suction cups have been used to deploy multi-sensor tags on large cetaceans [19], while time-releasing harnesses have been applied to sharks, mobulid rays [20] and leatherback sea turtles [21], and headcaps or epoxy-sealed mesh mounts have been used on pinnipeds [22]. Despite their importance, the development and validation of attachment techniques remain underreported in the literature, even though they critically influence data quality, animal welfare, and overall research success [23–25]. Although costs have declined, tracking technologies remain expensive, with individual tags often costing several thousand USD, which can strain budgets in resource-limited contexts and highlights the importance of maximising data yield per deployment and developing best-practice guidelines for their efficient and ethical use.

Perhaps one of the most morphologically and behaviourally diverse groups to which biologging devices have been attached are sharks [26], with at least 35 species from 12 families and five orders satellite tracked since 2010 [27]. For most large shark species, tag deployment is often facilitated through capture and physical restraint, allowing for precise placement and secure device attachment [28]. Capture-based methods typically employ netting, long-lining, set-lines or rod-and-reel fishing followed by the use of deck-mounted cradle systems, sling lifts, or in-water restraint of the shark during the procedure, either through tonic immobility or by securing it alongside the tagging vessel [28]. This semi-controlled environment enables the deployment of various tag types, including pop-up satellite archival transmitter (PSAT) tags, Argos satellite transmitters (PTTs, platform terminal transmitters) such as smart position and temperature (SPOT) transmitting tags (Wildlife Computers, Redmond, USA), and fine-scale archival data logging devices with sensors for depth, acceleration, and environmental variables [27]. These tags are most commonly attached by drilling through the first dorsal fin and securing the device with bolts. This method typically involves positioning the tag base on one side of the fin and passing bolts through the fin tissue, often with washers or backing plates to distribute load and reduce tissue stress or damage. Drill-based attachment provides high tag stability and is therefore widely used for long-term deployments; however, it requires close access to the animal and, in most cases, physical restraint [28].

Capture-based methods are not feasible for all large sharks [28]. The largest shark, for instance – the whale shark (*Rhincodon typus*) – presents challenges in this context due to its immense size, thick dermis, inability to be caught with hook and line techniques, and the fact that they cannot be easily restrained or safely hauled out of water using conventional methods. Although capture and restraint are possible, such opportunities are rare and logistically complex [29]. In these cases, tags can also be secured by drilling through the fin, but this requires opportunistic restraint and is only feasible in a limited number of locations globally [29]. These constraints have historically limited both the types of tags and attachment methods available for whale sharks, while simultaneously driving innovation in tag design and alternative deployment techniques.

Early in the tracking of this species, tag attachment primarily involved external tethers affixed with titanium or plastic dart anchors inserted into the subdermal connective tissue at the base of the first dorsal fin (e.g., [30]). Their generally docile behaviour allowed researchers to approach and tag sharks in the water without the need for restraint. However, this method can be prone to premature tag detachment due to improper implantation, hydrodynamic drag, entanglement, tissue rejection, or anchor failure, particularly during long-term deployments [31]. Anchors deployed intramuscularly can remain in place indefinitely, and tethers can attract large amounts of biofouling over long periods. Customised spring mechanisms that are ‘clamped’ around the dorsal fin to secure tags offer a promising alternative [24]. These attachments typically involve customised printed plates with textured surfaces, such as spikes or sandpaper [24, 25], mounted on stainless steel wire, often providing a more stable platform than traditional dart-based methods. Clamps can enhance tag stability and longevity, ultimately improving the quantity and quality of data collected. Considerable variation exists in clamp design – including materials, surface treatments, springs, and release link types – although formal descriptions in the literature are generally limited to brief summaries, with more detailed protocols reported primarily in studies introducing novel technologies [24, 25].

Designing clamps to attach to shark fins poses unique engineering and biological challenges, including accounting for the animal’s swimming biomechanics, dorsal fin shape and size variation, as well as potential differences in skin thickness and surface texture. Clamp-based attachments have been successfully applied to other large species, including white sharks (*Carcharodon carcharias*) [25], tiger sharks (*Galeocerdo cuvier*) [32] and sandbar sharks (*Carcharhinus plumbeus*) [33]. For these species, abrasive friction pads mounted on stainless steel clamps that conform to the dorsal fin without puncturing tissue have proven effective in securing biologgers for multiple days [25]. On tiger sharks, such clamps have enabled the collection of high‑resolution movement, depth, and multi‑sensor data over short periods [32]. One of the first clamp designs to be developed for whale sharks was deployed over 15 years ago [24]. The tag package was constructed from spring‑tensioned arms featuring ~ 15 mm spikes that latched into the cartilage of the 2nd dorsal fin, with the tag attached via a galvanic‑timed release (GTR). These clamps remained stable on the sharks for several hours after which the GTR allowed the tag to float to the surface for recovery once it corroded [24]. For most species, clamps have primarily been used for short-term deployments, remaining attached to the animal for only a few days. In contrast, for whale sharks, they are now widely used for long-term deployments intended to remain in place for several years (first placed on the 1st dorsal fin in 2012 to track long-distance movements, [34]). Longer-term deployments generally do not use a GTR and are designed to sit higher on the fin remaining stable and in place for months to years as opposed to days [34].

Long-term whale shark tagging efforts have consistently pushed biologging and telemetry boundaries [35], providing some of the longest (> 10,000 km e.g., [36]) and deepest (> 1900 m e.g., [37]) satellite tracking records of any shark species. Meanwhile, fine-scale behavioural tags have been used to address state-of-the-art topics such as estimating shark energetic costs of interacting with simultaneous human activities [38, 39] and three-dimensional linkages to biophysical environments [40], for instance. These insights have been used to help inform conservation, with satellite-derived movement data applied to estimate human threat exposure [41, 42] and examine potential relationships with oceanographic conditions [43, 44], which are in turn linked to projected distribution shifts under future climate scenarios [11, 45]. These research outputs have directly contributed to key policy instruments, including the 2024 Convention on the Conservation of Migratory Species of Wild Animals (CMS) resolution to mitigate ship strikes [46, 47] and the 2025 uplisting of whale sharks to Appendix I on the Convention on International Trade in Endangered Species of Wild Fauna and Flora (CITES) [48], demonstrating the clear value of satellite tracking for this species.

As tagging effort continues to expand in support of these research and conservation objectives, the ethical implications of clamping methodologies warrant careful consideration. In several species of shark, complex tag-related injuries have been documented, with some cases of seemingly permanent damage to the fin [49–51]. These injuries are most commonly associated with conventional tagging techniques that involve drilling into the fin and securing the tag with bolts. There is some evidence of healing after tag removal or natural tag migration of the device out of the fin over time [52]. However, the potential for fin damage caused specifically by clamps, as well as the subsequent healing, remains poorly understood by comparison. Attached tags can also change the hydrodynamic environment a shark experiences (e.g., increased drag) or induce short term alterations in behaviour, which are key considerations for both animal welfare and data collection [53]. The diversity of scientific teams, geographical study sites, and research objectives has led to considerable variation in tagging approaches (e.g., Table S2 in reference [41]). This complexity complicates efforts to standardise methodologies and optimise outcomes, hindering the community’s collective progress in understanding whale shark ecology and conservation in the most efficient and ethical way. With burgeoning use of biologging and telemetry devices [27], the field of marine biotelemetry and biologging is now in need of a systematic documentation of how researchers are applying clamp-based tagging methods, what challenges they face in real-world conditions, or how different designs and deployment techniques influence tag performance and retention, as well as animal welfare. This can also help determine whether long-term clamps – as well as the widely applied short-term clamps [25, 32, 33, 54] – could be adapted or improved for use on species other than whale sharks.

Despite the growing use of clamps to tag whale sharks, published information on best practices, deployment protocols, and scientific consensus regarding their design, advantages and drawbacks remains fragmented. To address this knowledge gap, we surveyed whale shark scientists from multiple international research groups actively or previously involved in whale shark tagging efforts worldwide. A range of clamp systems deployed across numerous tagging expeditions in known whale shark aggregation sites from almost two decades were assessed, with follow-up monitoring occasionally being available through photographic identification and, where applicable, tag transmission data. To harness this information, we conducted a semi-structured questionnaire survey to gather details on clamp tagging protocols, including the types of clamps used, attachment sites and position on the dorsal fin, deployment procedures and handling methods, post-deployment monitoring, observed complications, quality of resultant data, and perceived advantages and disadvantages of different approaches. The survey explored innovations in clamp design and emerging best practices aimed at reducing shark injury while maximising tag retention and data quality. We also present more detailed tag performance data and anecdotal evidence of clamp-based impacts from dedicated field expeditions in La Paz Bay, Mexico and from more than 90 tracked individuals tagged at four other sites. Through this methodological review, our primary objectives were (*i*) to assess the feasibility of deploying clamp systems during natural encounters compared to other available methods and pool information on how researchers are achieving this, (*ii*) to evaluate the physical clamp retention and performance of the attached tags over time, and (*iii*) to document any observable short-term impacts on the animals. Our study aims to help maximise success by identifying currently recommended design and deployment protocols learning from the collective experience of the research community. This can support acquiring the highest quality data to inform the conservation of endangered whale sharks and other large shark species amid global declines [55].

## Methods

A detailed, semi-structured questionnaire was designed to capture information on current and past whale shark tagging efforts, with a focus on clamp attachments. The survey was distributed via a targeted call to researchers currently active or previously involved in whale shark studies, working across known aggregation sites and within research institutions, non-governmental organisations and ecotourism operations globally. Researchers were identified through authorship on whale shark publications where telemetry and/or biologging was used and participation in the Global Shark Movement Project (GSMP, www.globalsharkmovement.org). It was circulated in early 2025 and remained open for approximately two months.

The questionnaire included a combination of multiple-choice questions, lists, and open-ended prompts to allow for detailed and nuanced responses (a complete list of questions is provided in Table S1), aiming to elicit both quantitative and qualitative data on clamp design, application protocols, and outcomes. Respondents were asked to provide details on the number and types of biologging devices deployed, the method used for attachment, and if clamps were used, the specific clamp designs implemented including materials, dimensions, and anchoring strategies. They were also asked to describe how and where the clamps were positioned on the dorsal fin and whether any specialised deployment aids – such as custom built arms used to hold and trigger release of spring tension (e.g., [25]) – were involved. In addition, respondents were asked to provide information on the effectiveness of clamp retention and the typical duration of deployments and quality of data. In most cases data quality was discussed qualitatively due to challenges in retroactively linking tag set ups with data relayed from past expeditions and the fact that tags can be programmed differently. Here we broadly considered quality data as: *i*) continuous transmissions (also known as uplinks) over an extended period for long-term tags (i.e., the device is transmitting regularly for months to years as per the device battery specifications), *ii*) consistent locations for long-term tags (i.e., transmissions are being successfully decoded into accurate locations without large or fragmented gaps in deployments allowing the reconstruction of movement patterns), *iii*) successful and consistent relay of collected data (i.e., transmissions are frequent enough to relay collated data that capture biologically relevant behaviours), and *iv*) successful recovery and download of biologically relevant behaviours from short-term tags. Most tag deployments fall along a spectrum; in the best case, the tag functions for its full programmed battery life, reports locations at the intended frequency, and successfully transmits all the desired data. In the worst case, the tag fails to transmit altogether. The closer a deployment is to the best-case scenario, the higher the resulting data quality.

**Fig. 1:**
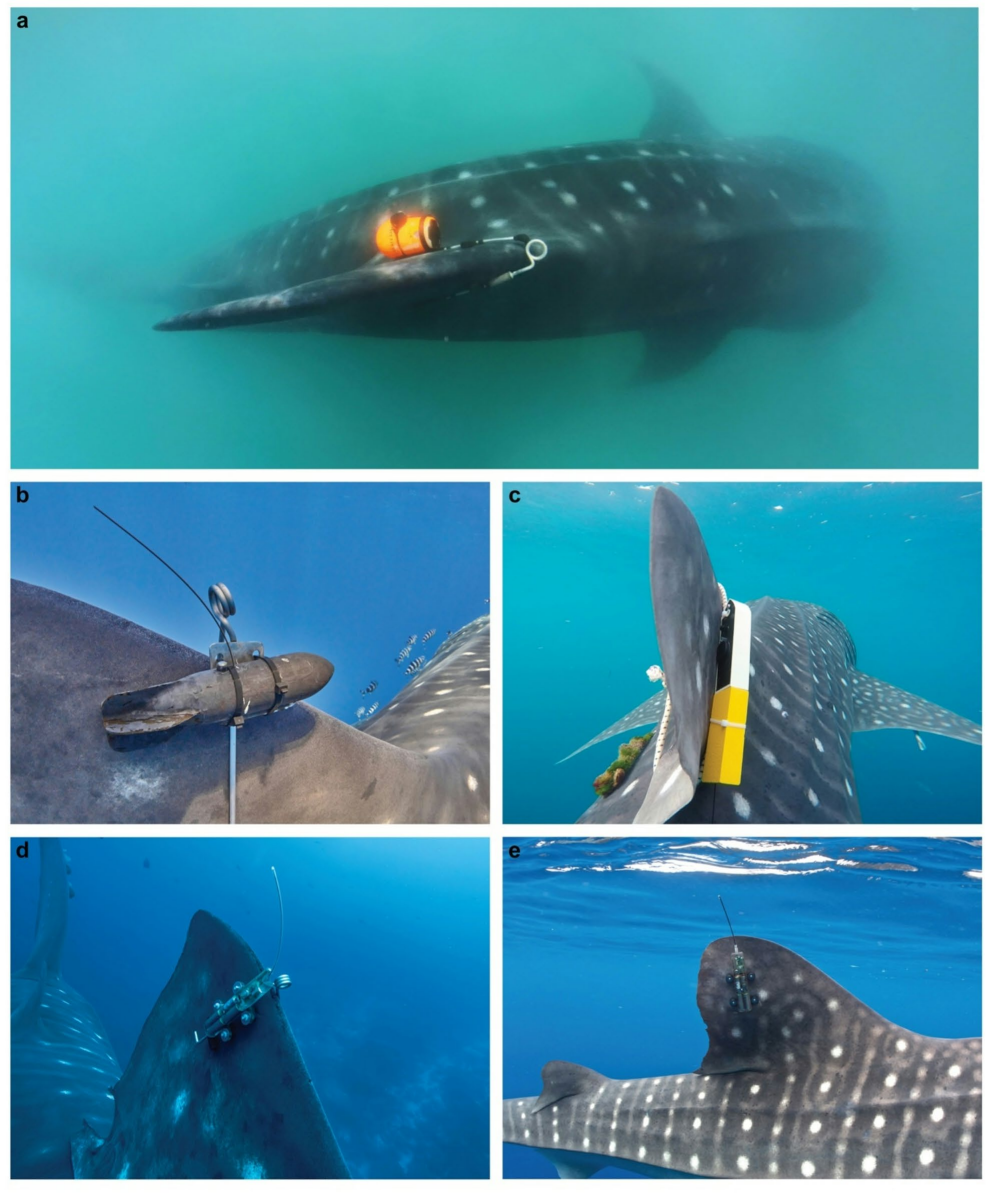
Examples of methods used to attach both long-term (i.e., satellite) and short-term (i.e., archival data logging) tags on to whale shark dorsal fins in **a** La Paz, Mexico (archival tag housed on a clamp, photo by Freya Womersley/Marine Biological Association), **b** Azores, Portugal (towed satellite tag housed on a clamp, photo by Jorge Fontes), **c** Saudi Arabia (archival tag housed on a bungee system, photo by Reef Ecology Lab), **d** Galápagos, Ecuador (satellite tag housed on a clamp, photo by Sofia Green), **e** Indonesia (satellite tag secured via drilling and bolting, photo by Mark Erdmann)

The survey was divided into two deployment categories based on tag type and intended duration: long-term and short-term deployments. Long-term referred to clamps housing satellite-transmitting and PSAT tags such as SPLASH10-346, SPOT-257, and MiniPAT-430 (Wildlife Computers; www.wildlifecomputers.com, Fig. 1b, d, e), among others. These tags estimate location using either the Argos satellite system – via Doppler shift when the animal surfaces – or light-based geolocation in the case of PSAT tags. Argos tags rely on a wet-dry sensor to detect surfacing and initiate transmission, while PSATs archive and summarise data and detach after a preset period. In addition to location, both tag types can collect environmental and behavioural data such as depth, temperature, and dive profiles over extended periods, though PSATs generally offer lower spatial accuracy for locations. Short-term deployments, by contrast, involved high-resolution archival tags such as PILOT [20] and DOME [56] tags, or Diary tags manufactured by Customized Animal Tracking Solutions (CATS; https://cats.is) and Daily Diary tags manufactured by Wildbyte Technologies, Swansea University [24] (Fig. 1a, c). These store detailed accelerometry, depth, and temperature data sampled at high frequencies (e.g., 10–40 Hz) and can be used to collect animal-borne video [54]. These tags do not transmit data but must be physically recovered and are typically deployed for less than a week. This allows fine-scale behavioural analysis, including body posture and kinematics, that is currently difficult to capture in long-term deployments [57, 58].

While both tag types were addressed in the survey, the questionnaire placed greater emphasis on long-term clamp design due to the specific challenges associated with extended retention. Short-term tags are usually straightforward to retrieve and do not require the same level of attachment robustness. Respondents who had not used clamp systems were encouraged to bypass those sections and instead share their experiences with alternative tagging methods in later parts of the survey. One focus of the survey was the potential physical impact of clamp attachments on whale sharks. Respondents were asked to report any evidence of fin damage, abrasion, or tissue trauma, and to comment on whether follow-up monitoring (e.g., through photo-identification) revealed any apparent short- or long-term impacts.

To validate and contextualise the largely qualitative survey findings, two additional quantitative assessments were undertaken. First, a complementary field assessment was conducted as part of whale shark behavioural research in La Paz, Mexico, in 2024. During this study, juvenile whale sharks were tagged using a variety of tag types and fin attachment positions. Although the expedition was not explicitly intended to test attachment performance metrics, it provided an opportunity to retrospectively examine clamp position performance and design impacts. Nineteen juvenile whale sharks (3.5 to 7.0 m estimated total length, TL) were tagged with SPOT-257s, SPLASH10-346s, SCOUT-Temp/Bathygraphs, SPLASH10-F-312s, or PSATs (Wildlife Computers), as well as short-term biologgers. Short-term biologgers were also trialled on three larger sharks in 2025 (making for a total of eight short term deployments). All long-term tags were deployed using clamps that shared the same base design, with only arm length and plate construction tailored to each tag type. Clamps comprised a 20 mm ring with two turns, a bridge distance of 45 mm at the head and 15 mm at the tail, and two 3D-printed plastic attachment pads fitted with 6 mm spikes on the dorsal surface and 12.5 mm spikes on the ventral surface. All tags were attached by hand while swimming alongside sharks and freediving.

Due to extensive surface use early in the deployments, followed by synchronous deep diving when individuals left the feeding aggregation site, deployment duration was not reviewed for the La Paz sharks to avoid confounding effects associated with track termination. Instead, transmission performance was explored in relation to tag placement on the fin, supported by follow-up photographic monitoring and assessment of the clamp impacts. To minimise confounding variables, only sharks tagged on the same day with identical tag models and configurations were selected for transmission comparison. This resulted in a sample of three individuals tagged with SPLASH10-346s and two tagged with SCOUT-Temp/Bathygraphs (Wildlife Computers). For these sharks, the number of transmissions and satellite passes recorded early in each deployment (first 45 days) were quantified to help account for potential positional slippage or other time-dependent effects. Only transmissions associated with a calculated location were included to limit the influence of ‘ghost’ transmissions. This design ensured that observed differences in transmission performance were most parsimoniously attributed to fin placement rather than tag model, clamp design, deployment method, biofouling, or clamp modification over time. While early transmission rates recorded at the same location using identical tags provide insight into the potential performance of different attachment positions, they do not account for individual variation in shark behaviour, such as diel or other patterns in surface use.

In addition to the field assessment, experts were asked to supply raw satellite data from sharks tagged using clamp designs that demonstrated both strong and weak performance, as well as those tagged using drilling procedures. For these submissions (*n* = 96 individuals), fin position was not consistently recorded during tagging expeditions; therefore, only clamp measurements and design features were assigned to each track. From these data, we calculated total track duration, number of days with at least one location recorded, and mean transmissions per day during the first 45 days, based only on transmissions for which a location was obtained (at least three transmissions are needed to estimate a location). To standardise earlier deployments, transmissions to the Kineis (www.kineis.com) constellation were removed. This dataset included two designs from expeditions in the North Atlantic and two from the Western Pacific. The two Western Pacific designs (Designs C and D) were deployed at the same location by the same core research team, allowing for direct comparison of two key designs while minimising confounding factors.

Track termination can occur for reasons unrelated to attachment performance or clamp design, including tag battery depletion, sensor or antenna failure, biofouling of the saltwater switch, changes in animal behaviour affecting surfacing frequency, or interactions with humans [59]. As such, track duration was interpreted as an integrative measure influenced by both attachment characteristics and external technical and behavioural factors, rather than as a direct proxy for attachment success alone. This also applies to the calculations of days with locations recorded and mean transmissions per day. Together, the two complementary quantitative assessments were designed to test the effect of clamp position on transmission potential (data quality, from La Paz expedition) and the effect of clamp design on overall track duration and transmission potential (data quantity and quality, from pooled expert data).

The expert questionnaire was voluntary and contained an opening paragraph explaining the purpose of the research. It required that participants consent to sharing information and knowledge with the understanding of how their data would be used. There were several limitations of this approach. Firstly, responses were self-reported and therefore subject to potential recall bias, particularly for historical deployments conducted many years prior. The level of detail varied among respondents depending on accessibility of past clamp designs, data availability and individual recollection. As such, some respondents did not fill out all sections of the survey and the sample size (*n*) reported in brackets after each result was based on the replies to a specific question. The survey may also reflect a degree of self-selection bias, with a greater likelihood of responses from researchers currently active in tagging projects or those with strong opinions on clamp design and performance. Consequently, results should be interpreted as indicative of broad trends and expert experiences rather than a fully representative or standardised dataset.

## Results

### Whale shark tracking overview

A total of 23 researchers submitted replies to the survey, which collectively represented the contributions of 57 scientists and conservationists globally. Although whale sharks were first tagged with satellite transmitters by researchers participating in the survey in 2001, most initiated tagging efforts around 2010 – the median start year – indicating a 15-year average history of satellite tagging in the field. Whale sharks have been tagged in at least 20 countries, including Australia, Brazil, Djibouti, Ecuador (Galápagos), India, Indonesia, Madagascar, Maldives, Mexico, Mozambique, Panama, Peru, the Philippines, Portugal (Azores), Qatar, Saudi Arabia, Seychelles, Tanzania, the United Kingdom (Saint Helena), and the United States. Australia was the most common tagging location, followed by Mexico and the Philippines. Across these efforts, more than 1,000 whale sharks have been tagged using a combination of long-term satellite transmitters and short-term archival devices (Fig. 1). On average, each individual respondent was involved in tagging approximately 60 sharks (*n* = 23 responses, which included some overlap between groups and expeditions).

The most commonly used method of attaching tags to whale sharks was via towed tethers longer than 1 m, used by 86.4% of respondents (*n* = 22 responses). Clamp attachments onto the dorsal fin were used by 81.8%, while shorter towed tethers (< 1 m or body-adjacent) were also common (68.2%, *n* = 22 responses). Other less frequent methods included directly drilling holes into the fin to attach the tag with bolts (18.2%), using elastic or bungee bands (13.6%), clamps with tethers attached (9.1%), harnesses (4.6%), and magnetic clamps (4.6%, *n* = 22 responses). To deploy clamps specifically, 86.4% of respondents swam alongside the shark using self-contained underwater breathing apparatus (SCUBA) or freediving and attached the clamp by hand (Fig. S1, *n* = 22 responses). Some teams (63.6%) used a deployment aid while swimming (e.g., a deployment ‘gun’, Video S1), while fewer used a vessel-based deployment aid or performed in-water static or drilling procedures where the animal is restrained (9.1% and 4.6%, respectively, *n* = 22 responses). When comparing perceived difficulty, respondents rated clamp deployment as substantially easier than drilling, with mean difficulty scores of 2.5 (*n* = 18 responses) versus 7.0 (*n* = 4 responses) out of 10, respectively (with 1 = ‘easy’, 10 = ‘difficult’).

### Methods of attaching tags

#### Dart-based methods

Tagging whale sharks using dart-based methods has involved a wide range of techniques, tag types, tether lengths, and outcomes over the last few decades (Table S2). Commonly, researchers use hand spears, Hawaiian slings, or pneumatic spear guns to deploy intradermal darts with tags such as PSATs (e.g., Wildlife Computers MiniPAT-430) and towed SPOTs (e.g., Wildlife Computers SPOT-425) attached. Tether lengths vary from 10 cm (for PSATs) to over 1.8 m for towed SPOT or SPLASH (e.g., Wildlife Computers SPLASH10-F-321) tags (Fig. S2). Many teams reported improved retention and reduced risk of premature detachment with shorter tethers and when the dart was well inserted into the muscle at an optimal location, which was identified as the flank near the base of the 1st dorsal fin aiming for a depth of 10–15 cm for pop-up tags and 15–25 cm for towed tags. Longer tethers usually > 1 m were needed to improve the number of transmissions for towed SPOTs or SPLASHs as they allow for the tag to float at the surface. It was noted that at slow and varied speeds (i.e., when a whale shark is feeding) buoyant tags move readily, which over time can rub against the shark’s skin causing abrasions that remain open until the tag releases. Longer tethers help to reduce this tag-body interaction; however, they are also associated with higher risks of entanglement with fishing gear, premature shedding due to drag, or tags being pulled out by other animals or humans. For example, in some sites other shark species have been observed interacting with tethered tags and ripping out the attachment, and whale sharks have been observed with towed tags where the anchor has torn the shark’s side from excessive drag forces. Whether tags were successfully attached using darts on the first attempt depended heavily on target shark behaviour during deployment (e.g., angle and motion), equipment setup (e.g., tether material, presence of swivels), the force used to deploy the tag, and the deployers experience with either hand spears or spear guns (Table S2). These factors varied with shark size, making precise anchor placement especially important for larger individuals. Thicker skin in larger sharks requires careful positioning and angle to achieve sufficient depth and required researchers to adapt methods and precision across size ranges.

Some approaches yielded quality long-term data (see Methods for quality definition), especially PSAT tags with short tethers, with retention exceeding one year. Others, such as towed SPOT or SPLASH tags with long tethers, typically lasted only a few months and occasionally failed to transmit due to hardware damage or detachment. For these deployments, if a long tether is used (> 1.5 m for SPLASH10-F, for example) researchers noted that considering the programmable constant depth settings – where the tag releases if depth remains constant for a set period – is important. For example, setting this feature to at least three days ensures that sharks staying at the surface for prolonged periods do not trigger this premature release in error (accounting for the length of the tether and range of depth change; if the tether is 2 m long and the depth range is ± 2.5 m then a shark staying shallower than 4.5 m for three days could trigger this). However, if the sharks are likely to stay in coastal waters this must be balanced with the possibility of detached tags washing ashore in this time frame before successfully transmitting any data. Researchers often adapted tagging strategies based on contexts, such as shark size, tagging location and expected behaviour of the animals. For example, techniques included using longer pole spears or specialised tips to improve penetration on larger individuals, often aiming for deeper muscle layers when feasible. Despite technical challenges – including variability in dart penetration, drag-induced detachment, and issues with depth-triggered releases – some teams increased the level of data quality by modifying standard equipment (e.g., inserting high-quality swivels into tethers to prevent coiling, using longer applicators (i.e., > 20 cm) or avoiding wire crimps to minimise points of weakness, Fig. S2). However, a few respondents abandoned spear methods altogether due to inconsistent performance and in some cases to avoid negative public perception of the practice (Table S2).

#### Drill-based methods

Drill-based attachment was the least common among respondents due to the logistical challenges involved in these procedures (e.g., the bespoke equipment required to restrain the shark and the need for SCUBA [29]). Nonetheless, several approaches have been trialled when conditions allowed. These have used either a battery-operated handheld drill from a vessel or swimming alongside a whale shark with a pneumatic drill connected to a low-pressure outlet on a standard SCUBA first-stage regulator and powered by a compressed gas cylinder (Fig. S1e). In Indonesia, for example, researchers take advantage of whale sharks entering fishing “bagan” – lift nets that target small teleost fishes – allowing them to work in-water within the net while the shark is temporarily restrained before release (e.g., [29]). More recently, new capture methods using mini purse seines, in collaboration with local fishers at inshore aggregation sites, have been developed with high success (Fig. S3). This is one of the very few opportunistic ways for a whale shark to be restrained in the wild, although it is likely not viable for larger individuals > 10 m (the largest shark tagged in this manner was 9 m TL). Smaller sharks have been noted to be more sensitive to the drilling procedure.

One team described using a template to drill four precisely placed holes in the dorsal fin with a cement masonry drill bit. Then, hypoallergenic nylon tubes were placed through the holes with ~ 3 mm protruding out on each side of the fin. The tags were then secured using stainless steel nuts and compressible rubber washers to accommodate natural fin growth over periods of 2 to 3 years (Fig. 1e). One respondent had attempted a similar method by approaching free-swimming whale sharks on SCUBA and performing each step individually when close enough. However, this was highly challenging, with some single deployments taking multiple weeks because the individual repeatedly dived and resurfaced, limiting opportunities to attach the tag. Only one research team has attached tags from a vessel after the shark was opportunistically restrained in fishing apparatus.

**Fig. 2:**
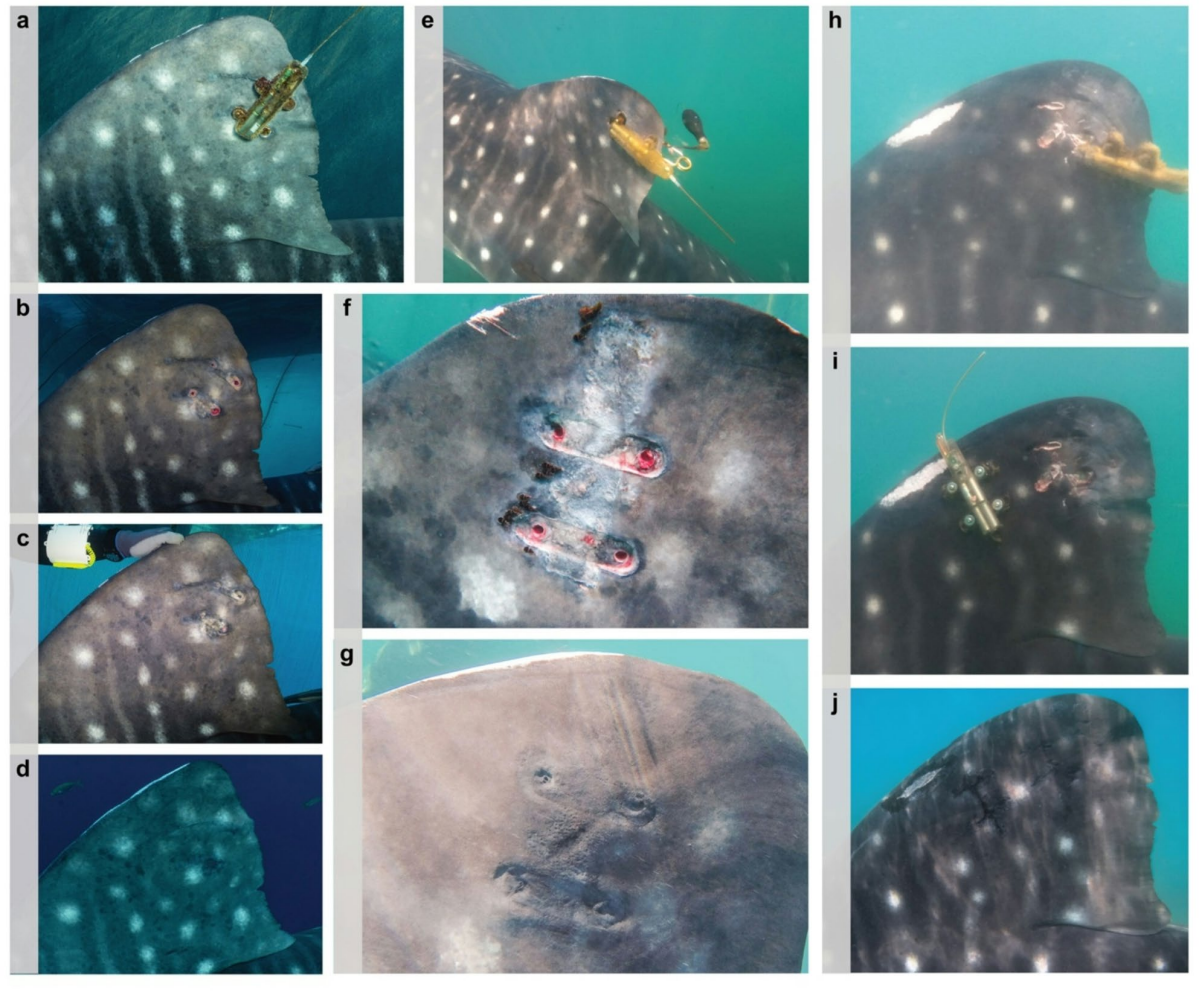
Examples of tagging related complications, injuries and subsequent healing showing **a** an individual tagged via drill-based methods with a Wildlife Computers SPLASH10-346 long-term satellite tag which migrated posteriorly (shifted towards the trailing edge) through the fin during the deployment. **b-d** show the same fin after the tag was removed, displaying the **b** day of removal, **c** three days post removal, and **d** 14 months post removal where full recovery is exhibited (photos by Mark Erdmann). **e** an individual double tagged with a SPOT-257 and MiniPAT-430 (Wildlife Computers long-term satellite and pop-up devices, respectively) with a clamp which slipped back over the top of the fin due to the initial position (c-3) and bridge distance that was too large (45 mm). This example also demonstrates biofoul accumulation possibly impacting tag performance (photo by Alberto García-Baciero). **f** shows another individual’s dorsal fin the day a tag was removed showing pallid surface skin irritation, open wounds and parasite invasion, which are completely healed **g** four months after the tag is removed (photos by Mark Erdmann). **h** shows another example of a clamp slipping with a Wildlife Computers SPLASH-346 tag (also showing the effects of biofoul) that has caused damage to the upper region of the fin due to spikes wobbling in the water flow. This tag was relocated **i** further forward on the fin and eventually removed showing full healing at **j** four months, though a scar in the shape of the attachment pads remained (photos by Alberto García-Baciero). Individual sharks are joined with a grey bar

Despite the methodological complexity associated with in-water drilling, teams have found it highly effective with potentially low impact over long timeframes after the initial invasive procedure. For example, tags typically remain in place for over two years, often until the battery expires, and can be opportunistically removed during routine resighting while sharks are feeding. In cases where removal does not occur, tags gradually migrate backwards due to drag and fall off naturally after approximately 3 to 3.5 years (Fig. 2a). Healing post-removal is typically complete within 3 to 14 months, with no observed fin deformation and minimal tissue damage (Fig. 2a–d, f, g). The technique has enabled the collection of high-resolution, multi-year tracking data, with some sharks successfully carrying tags and being re-tagged across successive deployments spanning over four years.

#### Clamp-based methods

Clamping was first used by researchers in 2007, with a median adoption year of 2018, eight years after the median for all tagging methods. Collectively, clamping methods have been employed on over 100 expeditions, with a mean of eight expeditions per respondent (*n* = 19 responses). Despite widespread tagging, fewer than half of respondents (43.5%) had used clamps for deploying long-term tags while nearly all (94.7%) had used clamps for short-term archival devices such as accelerometers and video tags (*n* = 23 including no response which inferred zero, Fig. 1). Almost a third of respondents had deployed more than 21 long-term clamp tags (30.4%), 17.4% had deployed between 11 and 20, and only three groups had deployed between one and 10 tags (1–5, 8.7%; 6–10, 4.4%, *n* = 23 including no response which inferred zero). Approximately half of those who used clamps had experience with double-tagging (e.g., deploying both short- and long-term devices simultaneously or deploying both SPOT and PSAT tags together). Although short-term clamp tagging was more common across responses, fewer sharks have been tagged this way. Over a quarter of respondents have tagged more than 21 individuals in this manner (30.4%), but fewer had tagged 11–20 individuals compared to long-term tags (8.7%; compared to 17.4%, *n* = 23 including no response which inferred zero). A wide range of shark size classes have been tagged using clamps. Over half of the surveyed groups reported tagging sharks between 3 and 12 m in length: 3–6 m (77.8%), 6–9 m (88.9%), and 9–12 m (61.1%, *n* = 18 responses). In contrast, smaller sharks (< 3 m) and very large individuals (> 12 m) were less commonly tagged, each reported by only 16.7% of respondents, likely due to their infrequent encounters (*n* = 18 responses).

Tag performance using long-term clamps varied widely. Most tags transmitted during the first seven days post-deployment, although some respondents saw low rates of transmission. For example, one group reported 51 of 75 long-term tags transmitting during the first week (Table S3). Transmission rates dropped notably beyond this initial window, raising concerns about early detachment, incorrect positioning or failure. The average maximum number of days transmitting was over 250 days, with some of these ongoing at the time the questionnaire was circulated in early 2025 (*n* = 11 responses). Of ~ 450 whale sharks tagged using clamps (Table S3), less than one third of these were seen again within the first week, though most respondents reported observing at least a few clamp tags on whale sharks within days of tagging (Table S4, *n* = 18 responses). Few respondents saw individuals again in subsequent weeks, and only about ten individuals with clamp tags were observed months after deployment (*n* = 18 responses). This shows an expected decline in re-sighting over time as individuals move away from where they were initially sighted.

### Clamp design, development and challenges

When asked what factor was most important in clamp development, the majority (66.7%) of respondents cited minimising impact on the animal (*n* = 18 responses). Tag retention was the primary concern for 16.7%, followed by data quality (11.1%, *n* = 18 responses). Researchers were evidently highly engaged in the clamp development process. Most respondents (94.7%) sourced clamp components off-the-shelf, with at least 11 teams working specifically with a single manufacturer (CATS, *n* = 19 responses). All purchased spring steel wire, while 61.1% also purchased attachment pads (*n* = 18 responses). Fewer researchers purchased deployment aids (27.8%), spikes (16.7%), or galvanic timed releases (GTRs, 5.6%, *n* = 18 responses). The majority of these off-the-shelf components were deployed unaltered, but many teams also modified parts before use. Modifications included glueing attachment pads in place (66.7%), bending or cutting spring steel wire (46.7% and 33.3%, respectively), and altering attachment pads (*n* = 15 responses). Several groups developed their own clamp components in-house, including the spring wire and attachment pads. While some teams found an effective design and continued using it without change, most iterated through multiple versions, with up to five different designs per group. Two groups stated they would not use clamps again for long-term tagging (citing concerns about potential fin damage and doubts regarding transmission reliability and tag retention over extended durations), while seven planned to further modify their designs. Seven other teams were satisfied with their current designs, and four were open to changing based on emerging data or peer designs. The most frequently reported issues among respondents included premature clamp detachment (70.6%), slippage into suboptimal positions (70.6%), and dorsal fin damage (58.8%, *n* = 17 responses). Fewer respondents reported poor initial tag transmission (21.4%) or failure to deploy clamps at all (5.9%, *n* = 17 responses). Failure to deploy was linked to sharks swimming too fast or too deep making it difficult to approach the individual, or team members struggling with the physicality of deployments. Respondents iteratively addressed some of these issues optimising clamp positioning and carefully developing hardware.

#### Long-term clamp positioning

**Fig. 3:**
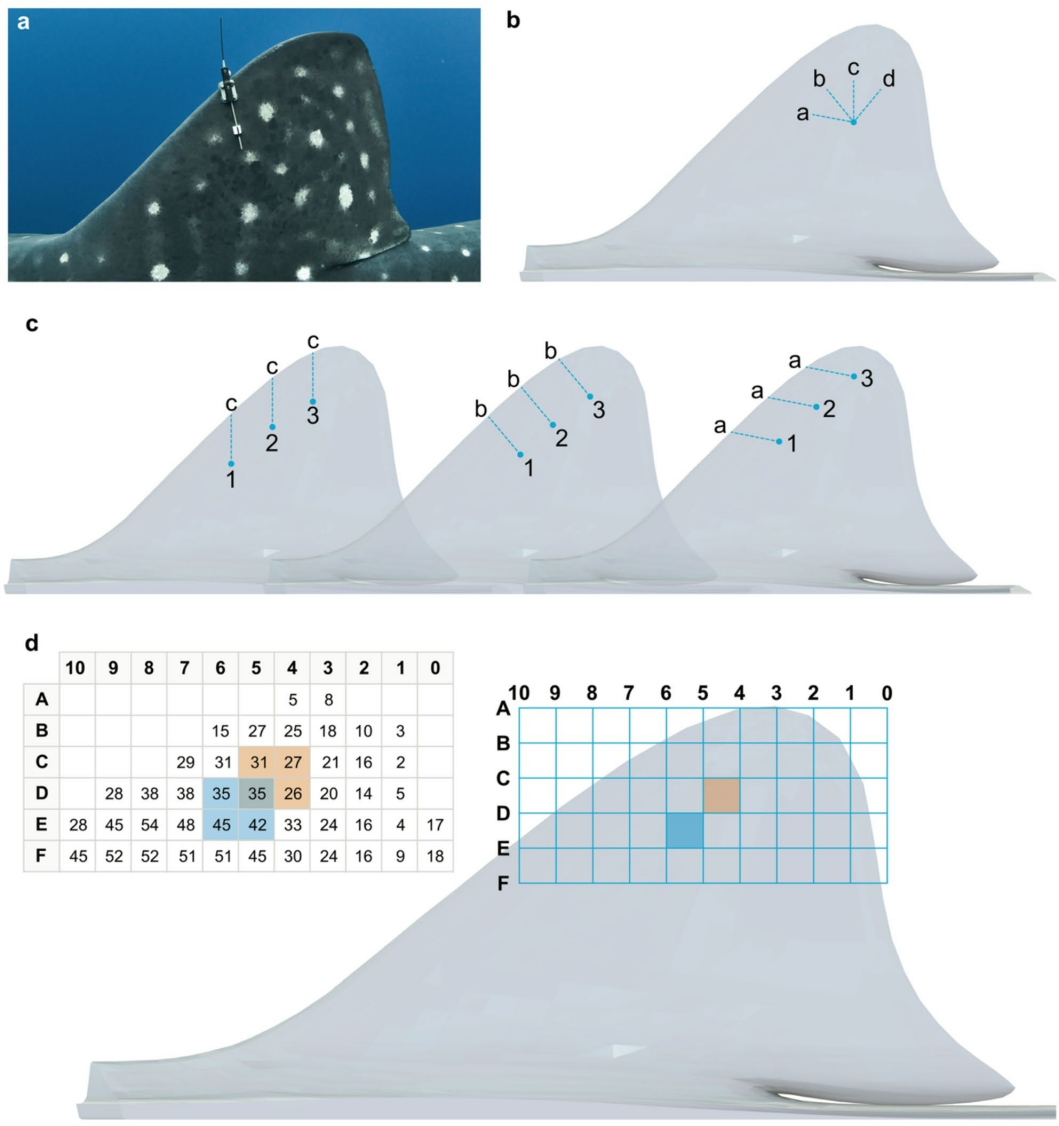
Tag and clamp positions used to guide questionnaire responses where **a** shows an example of a long-term clamp and satellite tag placed in position c-1 in the Azores (photo by Jorge Fontes), **b** and **c** show positions for the tag orientation and clamp orientation, respectively, **d** shows post-mortem measurements taken from a ~ 8.5 m total length male shark with highlighted areas averaging 39.25 mm (blue) and 29.75 mm (orange) thick. Estimated fin thicknesses for a ~ 7 m total length shark derived from 3D modelling and photogrammetry were 48 mm at position D6 and 51 mm at position E5. The fin model was created by DigitalLife3D and sourced from Sketchfab (www.sketchfab.com)

Among the primary tag placement configurations for long-term deployments, most respondents had the tag orientation running parallel to the clamp arms (Fig. 3a). Options b and c (50 and 90-degrees from the horizontal body axis, respectively, see Fig. 3b for tag positions) were the most trialled, each by 92.3% of respondents, followed by option a (≤ 10-degrees, 38.4%) and more rarely option d (130-degrees, 15.4%, *n* = 13 responses). When asked which configuration worked best, option b was rated highest (58.3%), followed by option c (33.3%) and option a (16.7%, *n* = 12 responses). Sub-categorisation of clamp positions revealed that b-2 was the most common (50-degrees positioned ~ 6/8 up the leading edge from the base of the fin, see Fig. 3c, 84.6%), followed by c-2 (90-degrees positioned ~ 6/8, 61.5%), b-1 (50-degrees positioned ~ 5/8, 46.2%), a-1 and c-1 (≤ 10-degrees positioned ~ 5/8 and 90-degrees positioned ~ 5/8, respectively, 38.5% each), and c-3 (90-degrees positioned ~ 7/8, 30.8%, *n* = 13 responses). Less frequently trialled were a-2 (≤ 10-degrees positioned ~ 6/8, 23.1%), a-3 and b-3 (≤ 10-degrees positioned ~ 7/8 and 50-degrees positioned ~ 7/8, respectively, 15.4% each, *n* = 13 responses). In terms of perceived performance, b-2 was rated as the most effective, with 50% of respondents identifying it as the best-performing configuration based on their field experience (*n* = 12 responses). Sub-options a-2 and c-2 were rated the best performing by 16.7%, while c-1 and c-3 were each noted by 8.3% (*n* = 12 responses; option a-1 is commonly used in short-term deployments and was ranked highly for performance but not included here due to the focus on long-term clamps).

Although positions c-1-3 were associated with high tag performance in terms of daily satellite transmissions, c-3 in particular, was frequently reported to suffer from stability issues, including sliding back on the fin and early detachment during dynamic conditions (i.e., where sharks are active surface feeding in rough surface waters). Positions b-1-3 were noted as still having solid tag performance with the additional benefit of stability and ease to deploy in dynamic situations, however, b-3 was also prone to slipping if not placed correctly in the first instance. Positions a-1-3 were reported as the most consistent in terms of physical stability; however, these were generally favoured by short-term tag deployments (*n* = 4 short-term responses, not included here). In all orientations respondents mostly designed clamps where the head rested directly against the fin leading edge, within 20 mm of the fin with a maximum of 50 mm away, but this sometimes came down to quick decision-making during deployments dictated by how closely the clamp fit the fin and how much time the tagging lead had to perfect positioning.

Field observations from La Paz Bay, Mexico corroborated the survey replies in relation to best performing tag positioning. One individual (5 m, male) tagged in position b-1 transmitted 17 times with 9 satellite passes over the course of 1.5 months (45 days), compared to another (6.1 m, male) tagged with the same device (Wildlife Computers SCOUT-Temp/Bathygraph) on the same day but in position c-3 which went on to transmit 638 times with 329 satellite passes during this time. Additionally, three further tags (Wildlife Computers SPLASH-346) were deployed on the same day in positions b-2 and b-3 onto three 4.5 to 5.5 m male sharks and programmed to transmit every other day. These transmitted 460 (b-3), 686 (b-2) and 990 (b-3) times with 93, 119 and 149 satellite passes, respectively, over the course of 1.5 months. One of the b-3 tags slipped after this timeframe (Fig. 2h) and stopped transmitting entirely until it was repositioned. These transmission frequencies are linked to the position of the wet-dry sensor. For example, theoretically repositioning a Wildlife Computers SPOT-420 tag from option b-1 to c-2 on a 12 m shark results in an approximate increase in vertical distance of 13 mm (37%) between the upper and lower wet-dry switches relative to the horizontal image plane and a 95 mm increase in the overall height of the uppermost sensor on the fin (Fig. S4).

#### Long-term clamp attachment hardware

Researchers used a range of clamp materials and configurations, the most frequent design – adopted by 92.3% – featured plastic plates or pads fitted with stainless steel spikes (*n* = 13 responses). Sandpaper was also common, used by 38.5% of respondents to enhance grip (*n* = 13 responses). Other methods included hybrid designs such as machined plastic plates with embedded spikes (7.7%), and steel frames soldered directly to the wire (23.1%, *n* = 13 responses). In some cases, spikes were also welded to the clamp wire itself. Typically, each side of the clamp was fitted with 1 to 4 pads, with pad placement adjusted according to clamp shape, tag type/orientation and shark dorsal fin width. Several groups modified their setups over time to problem-solve and improve outcomes.

Over half (58.3%) of researchers relied on pre-supplied attachment pads (e.g., from CATS), however, the rest fabricated their own with a range of materials used, including Polylactic Acid (PLA), Acrylonitrile Butadiene Styrene (ABS) and stainless steel (*n* = 12 responses). The most common approach involved 3D-printing pads and designing them to fit tightly around the clamp wire, which reduced the need for additional adhesives. While methods of glueing the pads in place were occasionally trialled, effectiveness varied. Some groups found adhesives inadequate in marine environments, especially on metal surfaces, whereas others reported success using ultra-strong adhesives (e.g., Araldite; Huntsman Advanced Materials GmbH, www.huntsman.com) to stop the pads shifting over time. Several groups developed a modular pad system using the same design for the non-tag side to simplify setup across different tag types. Across all designs, respondents highlighted the importance of achieving a precise fit, maintaining an even clamp pressure across the pad, and ensuring balanced spike placement; these considerations were deemed more critical than any single material or adhesive.

Spikes were a central feature across almost all clamp designs. Common options included stainless steel running shoe spikes, golf studs, and sharpened bolts, with lengths ranging from 6 to 30 mm. Most setups used two spikes per pad with up to six per side (where three pads are used), and the size and placement sometimes tailored to fin thickness. Variations included grinding bolt tips into flat facets, using sandpaper overlays, or testing alternatives like welded nails. For the flat facet approach, one team ground M5 hex head bolts to a point with 3 flat sides (made into pins for holding to the fin) before fixing lock nuts to the bolt and placing a rubber grommet over the top of the nut to minimise rubbing. Overall, plastic pads with spikes were reported as the most effective by 83.3% of respondents, followed by metal frames (16.7%) and combinations incorporating both spikes and sandpaper (8.3%, *n* = 12 responses). Designs lacking spikes tended to show poor retention rates and were frequently associated with early tag loss when used in long-term deployments (i.e., inferred from transmissions ceasing soon after deployment). Spikes generally rested against the skin without penetrating, but respondents observed that over the first few days post-deployment, they compressed further into the fin tissue demonstrating that clamps took time to settle into position.

**Fig. 4:**
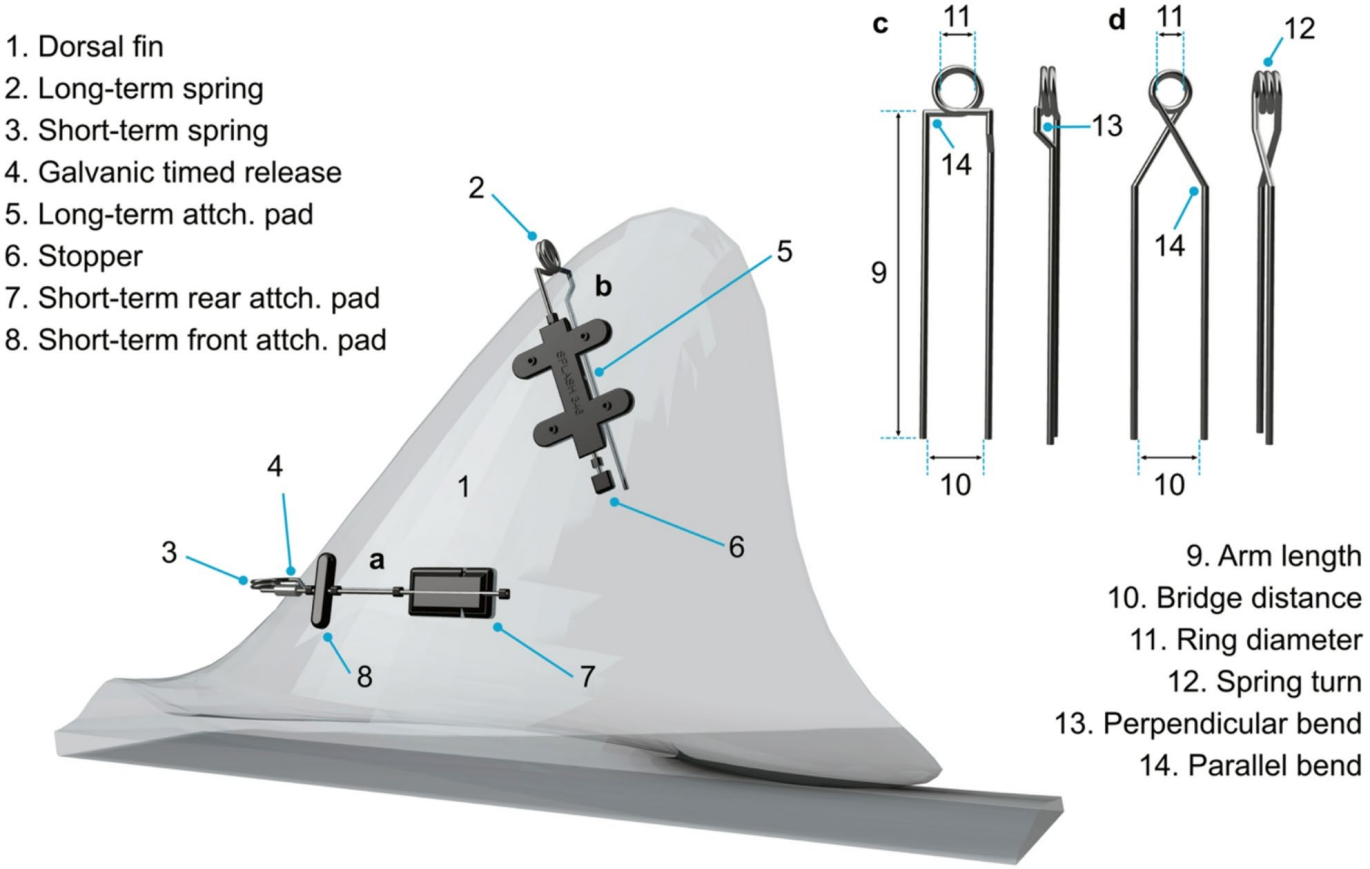
Schematic illustration of the currently recommended clamp position, designs and components. Long-term configuration in the upper fin (**a**) and short-term configuration in the lower fin (**b**). Square (**c**, first and second panels) and triangle (**d**, third and fourth panels) clamp designs are shown in detail. Numbered labels indicate key components. The fin model was created by DigitalLife3D and sourced from Sketchfab (www.sketchfab.com)

Clamp wire dimensions also varied across groups, with 1 to 3 turns of the spring coil, bridge distances ranging from 20 to 80 mm, arm lengths between 180 and 240 mm, and ring diameters from 15 to 30 mm (see Fig. 4 for measurement definitions). Those deemed the most successful by respondents had bridge distances ranging from 35 to 47 mm, arm lengths between 180 and 250 mm, and ring diameters from 20 to 25 mm. These depended on shark sizes with the upper range working well on sharks > 7 m TL and the lower range on sharks < 7 m. In general, small bridge distances (< 30 mm) relative to shark size were associated with scissoring and deforming the fin by being too tight. When asked how tight they felt their clamps were out of 10 (with 1 = ‘very loose’, 10 = ‘very tight’), researchers replied with a mean score of 6.3 (standard deviation (SD) = 2.2, range 3–9, *n* = 12 responses). Several groups manually exercised the clamps by pulling them open pre-deployment to remove or reduce tension at a given bridge distance (i.e., if the clamp rests naturally at 10 mm at the end of the arms it can be exercised to rest naturally at 30 mm). Most groups did not include any mechanism for the steel wire to detach over time, but one respondent described a coupling link designed to rust gradually and eventually lead to the entire attachment falling off.

#### Long-term clamp performance

**Fig. 5:**
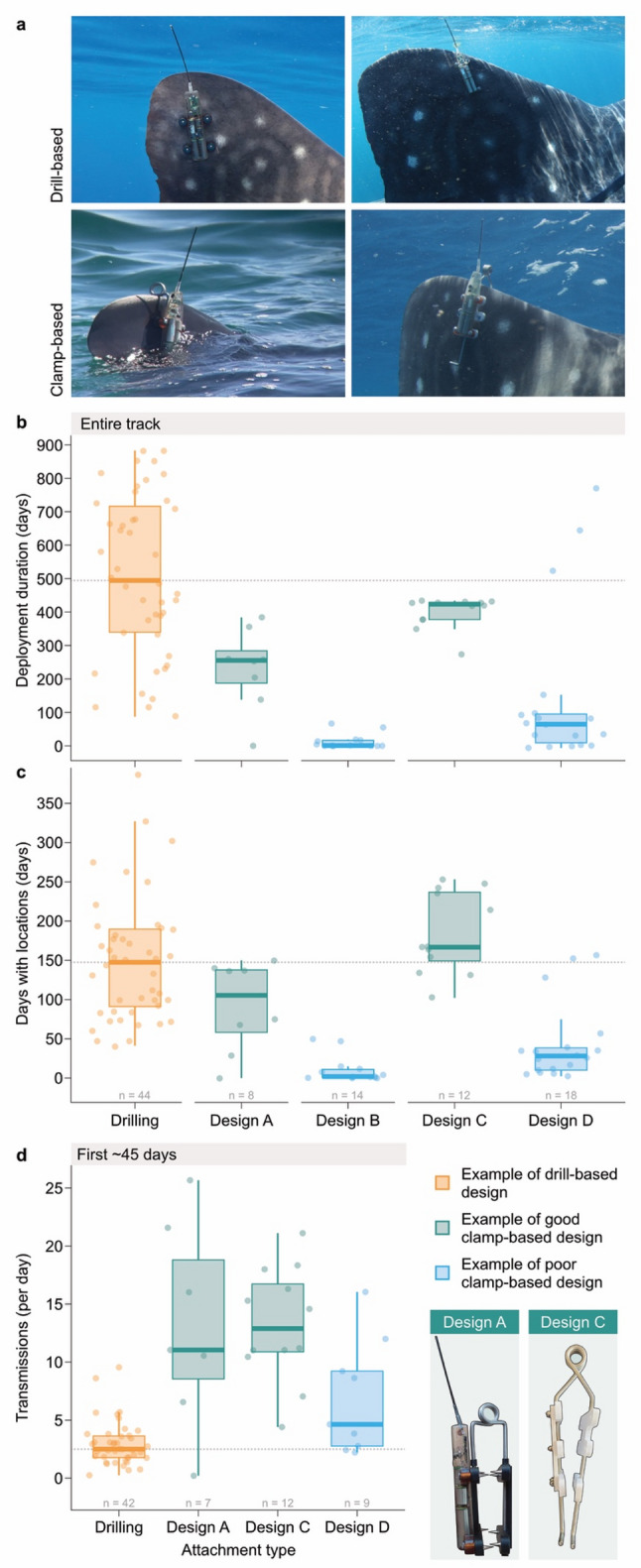
**a** Example of a drill-based deployment (top left panel photo by Mark Erdmann; top right panel photo by Rafael de la Parra) and clamp-based deployments (bottom left panel photo by Freya Womersley/Marine Biological Association; bottom right panel photo by Biopixel). **b** and **c** show total track duration and number of days with locations, respectively, for drill-based tags (orange), two examples of well-performing clamp designs (green), and two examples of poorly performing clamp designs (blue). **d** Shows mean number of transmissions per day calculated over the first 45 days of each track (tracks with cut offs > 5 days from 45 were excluded) for drill-based tags (orange), two examples of well-performing clamp designs (green), and one example of a poorly performing clamp design (blue). Design B was removed from this panel because too few tags reached the 45-day threshold. Grey dotted lines indicate the median for drill-based tags, and sample sizes are noted above each group on the x-axis in grey. Designs A and B were deployed in the North Atlantic, whereas Designs C and D were deployed from the same tagging location in the Western Pacific over consecutive trips. Drill-based tags were also deployed in the Western Pacific

Satellite-derived deployment metrics revealed that tags attached via drilling (*n* = 44 tracks) had the longest deployment durations (mean = 514.6 days, SD = 240.0, median = 498.0 days, Table S5) compared to four clamp designs. Drilled tags significantly outperformed two weak performing clamp designs (Designs B and D; post-hoc Dunn tests with Holm correction, adjusted *p* < 0.001), whereas two optimised clamp designs showed comparable performance. Design A (*n* = 8 tracks; square spring with two turns, 15–20 mm diameter, bridge distance of 30–40 mm at both head and tail, 12.5 mm spikes, and a single joined PLA plate on either side) averaged over 200 days (mean = 233.6 days, SD = 122.0, median = 257.0 days, Fig. 5b and Table S5) with some tags still transmitting. Design C (*n* = 12 tracks; triangle spring with three turns, 20 mm diameter, bridge distance of 47 mm at the head and 15 mm at the tail, spikes sharpened from bolts, and three modular ABS plates on either side) averaged close to 400 days (mean = 399.7 days, SD = 48.3, median = 423.5 days, Fig. 5a and Table S5), with some tags still transmitting. Neither Design A nor Design C differed significantly from drilling in deployment duration (post-hoc Dunn tests with Holm correction, adjusted *p* > 0.05), demonstrating the potential of well-designed clamp setups to achieve long-term deployments comparable to drilling.

In contrast, poor clamp designs produced substantially shorter tracks. Design B averaged less than 20 days (*n* = 14 tracks, mean = 14.6, SD = 21.8, median = 2.0 days; Fig. 5a and Table S5; square spring with two turns, 18 mm diameter, bridge distance of 37 mm at the head and 20 mm at the tail, 10 mm spikes, three PLA plates on either side, and the tag secured on an angled soldered plate which meant the entire body of the device protruded beyond the spring head and leading edge of the dorsal fin, Fig. S5), while Design D averaged less than 85 days (*n* = 18 tracks, mean = 84.8, SD = 144.0, median = 63.5 days; square spring with one turn, 30 mm diameter, bridge distance of 45 mm at the head and 15 mm at the tail, spikes sharpened from bolts, and two to three modular ABS plates on either side, Fig. S5). Design B was significantly shorter than Design C (post-hoc Dunn tests with Holm correction, adjusted *p* < 0.001). Although deployments occurred across multiple years, Designs C and D were deployed at the same tagging location helping to control for environmental and handling effects which may influence track termination. The precise causes of track termination cannot be determined and likely reflect multiple interacting factors (see Limitations and Future Directions section). Nevertheless, these results demonstrate that clamp attachments can achieve multi-year deployments.

While drilled tags showed the longest overall deployment durations, clamp designs performed as well as – or in some cases better than – drilling when considering the total number of days with locations recorded across the entire tracking duration and the mean number of transmissions per day during the first 40 to 45 days (Fig. 5c, d and Table S5). Design C significantly outperformed Designs B and D for total days with locations recorded (post-hoc Dunn tests with Holm correction, adjusted *p* < 0.001), and drilling outperformed the poorly performing clamp designs for this metric (Designs B and D; adjusted *p* < 0.001). Designs A and C also outperformed drilling when considering transmissions per day (post-hoc Dunn tests with Holm correction, adjusted *p* < 0.01, Fig. 5d and Table S5). Both the number of days with locations recorded and the mean transmissions per day are influenced by factors such as clamp position on the fin, which was not included here due to uncertainty in metadata collected during rapid or low-visibility encounters. These metrics are also influenced by satellite pass timing and individual shark behaviour (see Limitations and Future Directions section); however, the large sample size of drill-based tags suggests that the lower transmission rates observed for drilling may, at least in part, be related to attachment method. Taken together, these findings suggest that long-term clamp attachments represent a viable alternative to drilling, and that further optimisation could enhance performance now that baseline successful designs have been identified.

### Short-term clamp deployments

For short-term deployments, researchers used archival biologging tags to record fine-scale behavioural information at frequencies up to 40 Hz, monitor in-situ environmental conditions and collect video footage. Like long-term tags these devices can also be attached to free-swimming sharks during relatively brief encounters (typically a few minutes), meaning that tagged individuals do not require lengthy recovery periods, as is often the case for captured sharks. For example, sharks caught using hook-and-line techniques may take several hours to recover, whereas sharks tagged while remaining free-swimming can resume normal behaviour almost immediately [24, 56]. When focussing on fine-scale behaviours, clamps and attachments were designed for stability by minimising wobble and limiting data noise while placing the device at an angle that can be corrected for body position in post-processing [60], position a-1 being preferred. When stability was not a primary concern (e.g., some camera or environmental sensor configurations), tags were positioned to meet other objectives; some speed sensors need to face into the flow and cameras orientate to any desired target area (i.e., the head, [54]). As short-term tags often need to be recovered, designs incorporated a timed-release mechanism or some method to locate the animal again to be retrieved directly [61]. In these cases, unlike long-term clamps, the attachment can then also release from the fin since it is no longer of any scientific need. These devices were more often deployed using an aid (e.g., an applicator pole or a tagging ‘gun’, Fig. S1 and Video S1) compared to long-term clamps. The most common configuration for short-term clamps was the use of CATS data loggers attached to preset CATS springs which came in a range of sizes with an E8 magnesium GTR (approximate seven-day release time, www.underseareleases.com) built into one of the arms. Attached to these, teams have mostly trialled spikes and sandpaper integrated onto plastic pads, similar to those used in long-term deployments. In some cases, forward shark motion and wave action were enough to dislodge these devices (i.e., moving from position a-1 to c-3), emphasising the need for improving designs for stability.

As an alternative to the widely used CATS clamps, one design (Fig. 4) was developed and deployed onto eight whale sharks in La Paz Bay, Mexico (Fig. 1a). For this design, two spring sizes were used to test on sharks smaller or larger than 7 m. For smaller sharks, a 25 mm ring diameter was used with two turns of the spring coil and a perpendicular turn in the head of the arm with an E8 GTR. The bridge distance was 40 mm, and at an arm distance of 100 mm, an 170° bend was added with a total arm length of 300 mm (Fig. 4). The larger design had a ring diameter of 30 mm and a bridge distance of 45 mm. Two shallow grooves (~ 2 mm) were filed into each arm at 20 mm from the head to facilitate loading and firing from a deployment aid which was adapted from and inspired by CATS (Video S1, arm adapted to better house large clamp ring diameters). Attached to the springs were a set of bespoke PLA printed pads (Fig. S6) designed to prevent the front of the clamp from slipping upward and to provide ample stabilisation at the back of the clamp where the tag was situated (Fig. 4). Seven attempts using this design provided full 48-hour deployments, remaining in the exact deployment position while attached to the shark (one tag became dislodged after 26 h, possibly due to human interference). When tagging in deep water that exceeds the tag-tested pressure limits (often 500–2000 m), a machined float was attached to the adjacent side of the tag to offset the negative buoyancy if the entire unit became dislodged prematurely; a feature also used by several other respondents. The tag released at a programmed time integrated into the tag or timer connected to a time-released active cable tie. More than half of these sharks were resighted within weeks showing the clamps had shed or were in the process of shedding, with minimal fin damage remaining.

Besides clamps, groups have also used elastic bungee cords with GTRs (Fig. 1c), but these were challenging to deploy given that they must be pulled over the entire first dorsal fin. They also lack the stability needed to accurately classify fine-scale behaviours. Wooden clamp designs, similar to clothes pegs, have also been used by one group with grip enhanced by rough pads to increase tension and help retention. One team deployed towed tags using a 180 cm long polyethylene and monofilament harness. For this, a GTR and soft piece of rubber were integrated into the harness which was looped around the shark’s head allowing for adjustment to the animal’s girth, minimal over-tightening and entire removal post-deployment when the GTR link corroded. When deploying short-term clamps, respondents noticed that the whale sharks were more responsive, often banking, diving or speeding up compared to long-term deployments. This could be due to their often lower position on the fin, proximity of researchers deploying the device or larger clamp size. For example, one respondent noted that if extra pressure was applied to push spikes into the dorsal, the whale shark would react almost immediately and shake the clamp and logger package off forcefully. Some groups also attempted to attach short term tags to the pectoral fin using off-the-shelf plastic spring clamps, however this was said to be more challenging than dorsal fin deployments.

### Clamp-related injuries and subsequent healing

Clamp-related injuries were frequently attributed to incorrect placement on the fin, excessive tension, or direct mechanical damage from spikes and sandpaper. Incorrect clamp position on the fin can lead to it being overly tight if placed too low (i.e., pads pressing deep into fin tissues, Fig. S7a) or too loose and the attachment slipping if too high (i.e., spikes scratching and abrading the surface layers of skin, Fig. 2e, h and Fig. S7d). Damage due to compression was not immediately noticeable and tags were initially reported to fit well. However, the impacts of compression were seen after three or more days which many researchers did not have the opportunity to witness (Fig. S7a, b). Larger bridge distances (> 45 mm) were associated with the long-term clamps slipping into sub-optimal positions compromising tag performance and potentially damaging the fin via spike movement (Fig. 2e, h). Wounds can also result from placing a clamp on a shark that is either too large or too small for the design. Tissue embedding of the tag was a commonly noted issue, suggesting that clamps may generally be deployed too tightly, at least for some areas of the fin. In terms of short-term clamp related injuries, GTRs and the reactive steel wire have been seen to cause tissue damage (Fig. S7c). To alleviate this, one method trialled by two respondents was to add shrink tube or electrical tape to the wire itself. In addition, these clamps were reported to dig into the fin both during and after deployments, a problem that was reduced by securing pads towards the end of the wire. All injuries where sharks were sighted again showed full healing in a matter of months (Fig. 2).

In La Paz Bay, Mexico, one 4.5 m male shark was tagged with a SPLASH10-346 on the 13th December 2024. The shark had a partially amputated upper caudal fin and several other minor abrasion wounds. The clamp was placed in position b-3, and when the shark was resighted approximately one month later (17th January 2025), it had slipped over the arch of the fin and was causing damage due to spike movements in the flow (Fig. 2h). Ten days later, the tag was removed, cleaned and replaced on the same individual in position b-1 (26th January 2025, Fig. 2i). It remained in this position for approximately one month whereupon the shark was resighted (21st February 2025). On this occasion, the injuries from the initial tag position had begun to heal, although a small hole remained open in the dorsal fin. The tag was deemed too tight for its second position on the fin (i.e., bridge distance < 30 mm and tension designed for a higher position) and was therefore removed, leaving an open compression injury approximately 10 mm deep on either side of the fin surrounded by an area of pallid outer skin. Approximately one month later (18th March 2025), the abrasions from the first tag position were almost entirely healed, with the hole now closed, but the compression wound from the second tag position remained open with a moderate reduction in perimeter and a small reduction in depth. When the shark was resighted approximately three months later (11th June 2025, Fig. 2j), all wounds had fully closed and only a darker skin area remained where the second tag position had been.

In regions with long historical tagging operations, some previously tagged whale sharks have been observed with bent over dorsal fins resulting from long-term clamp deployments. Clamps can also lead to indirect injuries if people try to remove them and harm the animal in doing so; in one study site, a shark was injured by a vessel propeller as fishers attempted to remove the tag. Clamps can also get bio-fouled with a range of marine organisms (Fig. 2a, e, h). Biofouling is accelerated in warm shallow waters where some whale sharks are known to aggregate. Biofouling can severely inhibit tag performance by overgrowing the wet-dry sensors (Fig. S4) preventing them from drying out when the tag is in air. In the worst cases, fully operational tags with ample battery life can stop transmitting entirely. This is unlikely to be as much of an issue in cases where sharks are not aggregating but rather passing through tagging sites with some respondents reporting that applying antifouling paint to the tags did not change retention or transmission times.

## Discussion

Bringing together insights from whale shark researchers globally revealed valuable commonalities in the field of tag attachment development and deployment. The findings indicate that rapid innovation is already underway, particularly in emerging solutions for both long- and short-term clamp tagging. The widespread use of off-the-shelf (e.g., CATS) hardware illustrates the benefits of shared platforms and unified designs, while also revealing the reliance of many projects on a small number of suppliers. At the same time, there is growing interest among researchers in developing and customising their own equipment. Future development should emphasise open-source designs, iterative testing across study sites, and transparent reporting of both successes and failures. Such collaboration is especially important given that clamp-related knowledge is currently fragmented across research groups, limiting the collective capacity of the field to converge on best practices and solutions which minimise impact on the animal, which was a key consideration for most whale shark researchers.

Despite ethical concerns among respondents, clamp related physical injuries were still apparent. The 3Rs in animal ethics can be used to consider this problem by applying the principles of Replacement, Reduction, and Refinement [62]. In the case of tagging whale sharks, full Replacement (i.e., substituting animal use altogether) is not feasible because direct tracking remains essential to understand important behavioural, ecological and conservation questions for this species. However, clamp-based tagging represents clear progress within the other two dimensions, while still allowing for partial replacement through complementary less-invasive methods. Reduction can be achieved when each tag deployment yields more useful data of higher quality, thereby decreasing the overall number of animals that need to be tagged. Our survey suggests that clamp systems can extend attachment durations and accommodate a broader range of tag types compared to darted systems, while achieving data quality comparable to drill-based methods. The ability to restrain free-swimming whale sharks is rare, challenging and likely only possible in a handful of opportunistic scenarios, limiting the practicality of drilling. Moreover, the drilling procedure itself is inherently more invasive. Long-term tethered tags may also increase drag relative to clamp-based methods, potentially altering tagged animal behaviour over extended periods, especially if biofouling accumulates on the attachment. At present, therefore, clamp-based tagging represents the most suitable option across field situations to support the principle of Reduction where possible. Broader adoption of best-practice clamp approaches such as coordinated sharing of successful designs and open reporting of failures would also prevent unnecessary duplication and help reduce the total number of interventions across projects.

Although clamps may initially seem less invasive than drill-based methods because they avoid creating multiple holes through the entire fin, our findings show that Refinement is still needed. Problems such as over-compression, slippage, and abrasive surfaces can cause temporary injuries when using clamps. Whale sharks are known to heal well from complex injuries [63], but practical steps should be taken to minimise these impacts. These steps include tailoring clamp size and spring tension to size-specific fin morphology, using less-abrasive, biocompatible materials – avoiding sandpaper or other highly abrasive surfaces – and employing polymers such as nylon (PA12; smooth, chemically inert), polyurethane (flexible and soft), or polyether ether ketone (PEEK; durable and chemically stable in contact with tissue). Sharing insights across the tagging community can help ensure injury inducing designs are minimised. Additional measures include incorporating timed-release or breakaway features particularly for short-term deployments, and monitoring healing systematically through photo-identification (photo-ID) networks where possible. Programmes such as Sharkbook.ai provide opportunities and detect long-term tagging outcomes without additional handling. These platforms can be used not only to assess impacts of tagging, but also to complement data obtained from electronic tags [64, 65]. Photo‑ID and telemetry studies have successfully combined catalogue‑based identification with movement data to reveal site fidelity, connectivity and range shifts in whale sharks and other species [66, 67]. Although complete Replacement is not currently feasible, photo-ID and other partial alternatives can meaningfully complement clamp-based tagging, reducing reliance on invasive methods where appropriate. By embedding clamp use within the 3Rs framework, whale shark research can generate high-quality ecological data while also meeting the ethical obligation to minimise harm. To assist in the process of Refinement, the following section synthesises common themes amongst the survey responses to outline a blueprint for clamp design and deployment protocols which can be further improved in future with increased testing, reporting and collaboration.

### Clamp design and deployment synthesis

When deploying clamps with long-term satellite tags attached, the survey results suggest that the tag antenna and wet-dry sensors be positioned between options b and c and the clamp itself be positioned between options b-2 and c-2 (between 50 and 90-degrees, positioned 6/8 up the leading fin edge). In doing so this balances the trade-off between clamps slipping backward and tag transmission performance and data quality. This positioning is the most practical for deployments involving active animal behaviour given the increased margin for error when attaching. More vertical positions offer valuable benefits if deployed correctly but can require a greater degree of accuracy to ensure a stable fit or opportunity to adjust the clamp position in a second interaction with the tagged shark. As well as the angle on the fin, to minimise drag and therefore maximise longevity, the tag should not protrude excessively above the natural curve of the fin leading edge, this should also help with the durability of the antenna/transmission effectiveness. Protrusion beyond the fin leading edge is the most likely explanation for the short track durations observed in Design B, given that the clamp dimensions were comparable to those used in other deployments. When deploying in sites where whale sharks remain in shallow sunlit waters for extended periods, tags and pads should be painted with anti-foul paint or an ablative silicone-based coating like Propspeed™ (www.propspeed.com), ensuring that no paint is applied to areas in direct contact with the shark’s skin. If tagged sharks are expected to undertake deep dives soon after deployment, anti-fouling paint is less critical and should be avoided to prevent potential damage to the outer fin tissue.

The survey outcomes suggest two main spring designs for long-term satellite tag deployments built from 5 mm diameter Austenitic (300 series, i.e., 302) stainless-steel wire. The first has two 90-degree parallel bends at the top of the spring head (here termed ‘square spring’, Fig. 4c) and the other has a single 60 to 80-degree crossover at the spring head followed by two ~ 135-degree bends on each arm (here termed ‘triangular spring’, Fig. 4d). For the square spring, two turns are suggested with a ring diameter of 20 mm (minimum 15, maximum 25 mm). Based on plastic pads adding 2.5 to 5 mm of additional contact to each side of the clamp, a bridge distance of 40 mm at the head (i.e., where the ring meets the arms; minimum 30 mm, maximum 45 mm) and a minimum of 30 mm at the tail (i.e., where the spring ends; maximum 50 mm) is suggested while the spring is in a resting position. In this configuration, < 30 mm at the tail appears too tight and can lead to complex compression injuries with the additional 5 to 10 mm added by the attachment pads. A shark of ~ 8 m total length has an approximate fin thickness of 40 mm positioned at 75 mm along option b-2 (Fig. 3d D6) and 45 mm at 125 mm (Fig. 3d E5); therefore, if pads/spikes are placed 125 mm down the clamp, then they should consider the compression on ~ 45 mm of tissue thickness in this area. Based on approximate thickness measurements clamps should aim to apply tension across 30–40 mm of fin thickness (Fig. 3d). Any more than 15 mm of moderate clamp tension combined with a further 10 mm of spikes appears to cause damage to the fin, with the trade-off between tag longevity and damage to the animal, albeit temporary. A perpendicular bend should be placed at the head of the spring such that the two arms lie on the same axis. Arm lengths between 180 and 220 mm are suggested with longer arms allowing the attachment pads to be positioned further down on the spring for larger sharks (Fig. S8).

For the triangular spring, three turns are recommended with a ring diameter of 20 mm (Fig. 4d; note that a greater number of turns reduces spring tension, and a two-turn configuration has not yet been trialled in the triangular design). The distance between the spring head and parallel ends should be 40 to 50 mm, with the bends at 135 to 145-degrees. Based on plastic pads adding 2.5 to 5 mm of additional contact to each side of the clamp, a bridge distance of 40 mm where the arms bend (minimum 35 mm, maximum 45 mm) is suggested. Arm lengths between 180 and 220 mm are also recommended. For sharks > 4 m in total length using the square configuration, if the steel legs are straight and the bridge distance at the head is < 40 mm, then the top spike section will meet the fin first. This requires smaller spikes and narrower plastic pads at the top to ensure the bottom is in contact with the fin (Figs. S8, S9). Conversely, if the bridge distance is > 40 mm, then the top section will need larger spikes and a thicker plastic section to grip the fin (Figs. S8, S9). Based on field observations, a 40 mm bridge distance balances this and no variation in pad thickness is needed. For both square and triangle springs, one pair of stabilising spikes would ideally be positioned at the top (50 to 150 mm from the spring head) and one positioned below (50 to 100 mm from the top spikes), with an additional optional middle section between the two. These can be attached onto 3D-printed or machined plastic plates, which should be partially secured into place to avoid significant movement up and down the clamp arm. They should allow movement around the wire to adapt to the fin shape. The spikes should be 12.5 mm long (minimum 5 and maximum 15 mm) and made of stainless steel where possible or machined from hypoallergenic nylon. At the tail end of the spring, 90-degree turns or plastic stoppers can be used to prevent the tip digging into the fin. To secure the pads in place, we suggest they are printed with a 5.2 to 5.4 mm diameter hole depending on plastic used and printer settings, with the aim of fitting closely to the wire but being able to rotate laterally to meet the shape of the fin. For extra support these can be secured from moving up and down the clamp with stoppers applied directly to the steel with ultra-strong epoxy (Fig. 4, number 6).

**Fig. 6:**
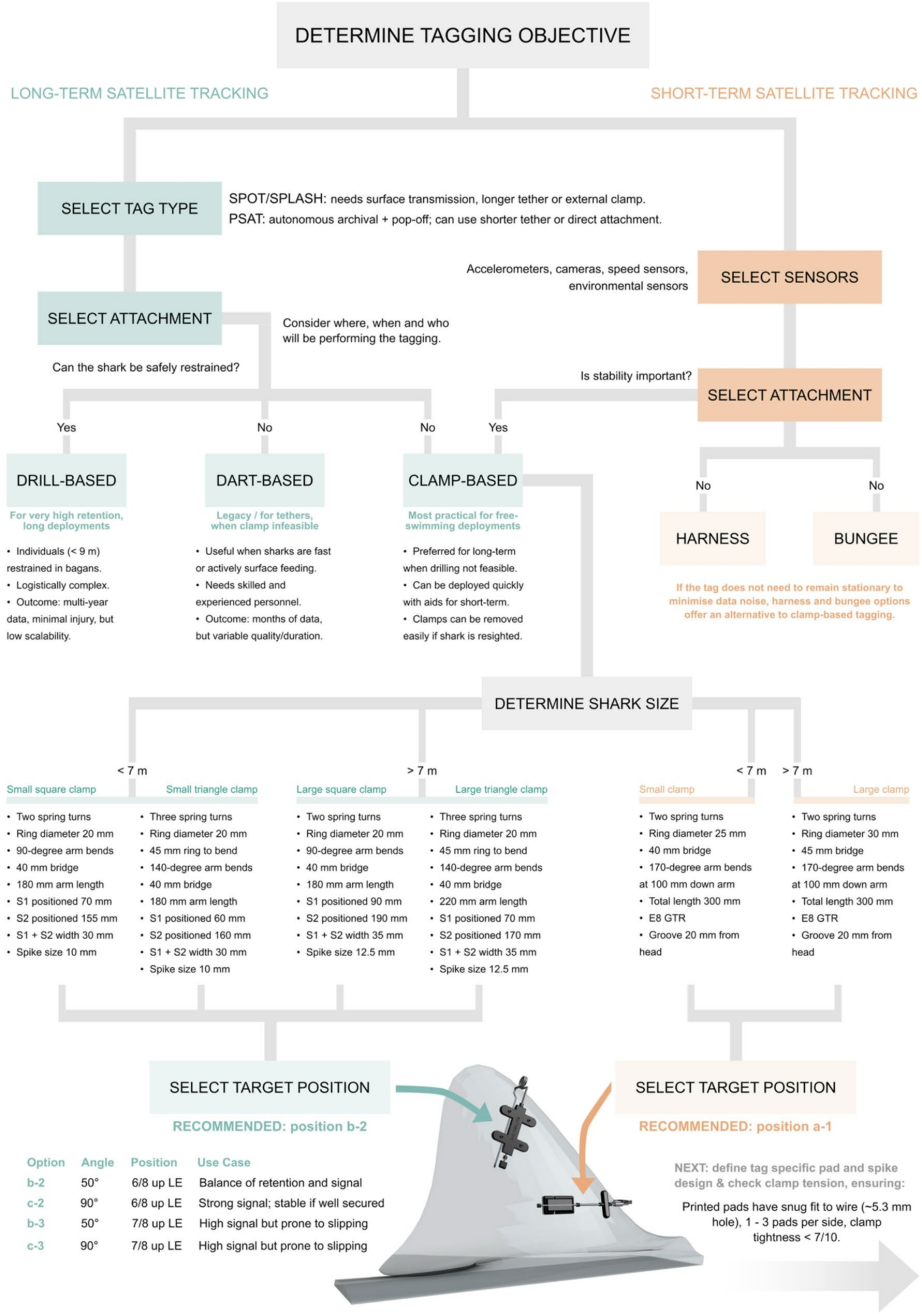
Recommended whale shark tagging decision making flow diagram. S1 refers to the location of the 1st spikes (see Table 1) and S2 the 2nd spikes. LE refers to leading edge of the first dorsal fin. Case study: a novice team (first time tagging whale sharks) aims to tag adult male sharks (9 m) in a new site where individuals pass through swimming slowly and close to the surface. The aim of the study is to understand the long-distance movements of this demographic with a good degree of resolution. Decision making: Because of their size these sharks cannot be safely restrained, especially as it’s a new tagging site with a novice team. Location resolution is a priority so deploying long-term SPOT (Wildlife Computers, or similar) tags via a clamp is the recommended approach for the study objectives. As the sharks are likely to be > 7 m, a large square clamp is needed, and due to the novice team deploying the tag, there is a risk of not securing the tag correctly, therefore position b-2 is recommended to balance retention and transmission strength. Case study: an experienced team aims to tag juvenile females (5 m) in a newly discovered coastal aggregation site to record their feeding behaviours and establish if they are feeding at depth. Decision making: A high-resolution tag is needed to pick out fine-scale feeding behaviours incorporating sensors that can record pressure, accelerometry and potentially cameras for validation. A high level of stability is required to ensure the signal noise is kept to a minimum when analysing the accelerometer data and assist in the identification of key behaviours, therefore a clamp-based attachment is recommended. The target females are < 5 m so a small short-term clamp deployed in position a-1 is recommended to align the tag with the shark’s body axis to help obtain accurate pitch and roll readings, which are useful for interpreting the different feeding behaviours. The expertise of the tagging team can help to position the tag in this manner

Overall, the consolidated survey responses indicate that both the square and triangle clamps can be used for whale sharks ranging from 4 to 14 m in total length, with adjustments made to the pads and spikes for different sizes where needed, the most practical being two size groups, above and below 7 m. This assists with manufacturing and shipping costs where clamps are being made to order, as the position and thickness of the attachment pads can allow for size flexibility. With the aim of providing equal tension at both the top and bottom of the clamp, a rough guide can be used to best determine the recommended resting distances between the plastic pads based on the estimated fin thicknesses between positions b-2 to c-2 (Figs. 3d, S8 and Table 1). Where clamp pads are difficult to source, then bridge distances can be altered for size-based measurements in Table 1. For example, if the pads lie flush against the spring arm (providing no additional compression distance) then a distance of 30 mm for sharks < 7 m in total length or 35 mm for sharks > 7 m should allow for a suitable fit. When planning whale shark tagging expeditions these design and deployment best practices should assist in decision making (see Fig. 6 for decision making flowchart).

**Table 1:** Suggested clamp dimensions based on compiled survey insights and contributed field experimental data. All measurements are in millimetres (mm) except for whale shark ‘Size’ which refers to whale shark total length in metres (m). Bridge refers to the bridge distance (Fig. 4), Spike (1st, 2nd ) refers to the distance down the clamp arm from the head where the spikes are located. The 1st and 2nd spike columns refer to the distance between the plates where the spikes are located, and the Spike column refers to the length of the spikes. See Fig. 4 for distance definitions and Fig. S7 for visualisation of rows two and five

Type	Size	Bridge	Spike (1st, 2nd )	1st spikes	2nd spikes	Spike	Arm length
Square	< 7 m	45	70, 155	30	35	10	180
		40*	70, 155	30	30	10	180
		35	70, 155	35	30	10	180
	> 7 m	45	90, 190	35	40	12.5	220
		40*	90, 190	35	35	12.5	220
		35	90, 190	40	35	12.5	220
Triangle	< 7 m	40*	60,160	30	30	10	180
	> 7 m	40*	70, 170	35	35	12.5	220

*Currently recommended design

This blueprint can also be broadly adapted to deploy long-term clamps less invasively for other species like white sharks, tiger sharks and basking sharks, *Cetorhinus maximus*, or indeed other fins, such as the pectoral fin based on the general relationships between fin sizes, desired compression and fit, and resultant clamp measurements reported here for whale sharks.

### Limitations and future directions

One of the key limitations of assessing the performance of clamp-based tagging of whale sharks using questionnaires derived from previous tagging expeditions is that researchers are constrained by the information recorded at the time of deployment. In some cases, neither the position of the tag nor the specific clamp design deployed on an individual shark was documented, making it difficult to fully quantify how design and placement influence tag performance in terms of data quantity and quality.

Additionally, when attempting to quantify performance, pooling information at a global scale introduces several confounding factors unrelated to clamp design or position that can influence performance metrics. These include, but are not limited to, location-specific whale shark behaviour, satellite pass timing, and exposure to human activities. For example, whale sharks aggregating at feeding sites may exhibit diel surface-use patterns that coincide with satellite overpasses, resulting in higher transmission rates than at sites where sharks are not feeding or where satellite passes do not align with surfacing behaviour. Similarly, if individuals are tagged in areas characterised by frequent deep diving or prolonged use of sunlit surface waters that promote biofouling, tags may appear to have suboptimal performance or short deployment durations that are unrelated to clamp design. High levels of human activity can further confound interpretation, as tags may be removed, sharks may be accidentally captured in fishing gear, or individuals may be fatally struck by vessels; events that have all been documented by whale shark researchers who took part in the survey. Under such circumstances, it is not possible to accurately attribute observed tag performance metrics to clamp design and/or attachment position alone.

To help address this complexity, we examined clamp position using sharks tagged on the same day, at the same location, with the same device and attachment setup, thereby limiting the influence of location-specific behaviour and satellite pass timing, although individual behavioural variability remains. We also focused on case studies that either performed particularly well or poorly, providing illustrative examples of clamp designs with potential for successful long-term deployments compared to those less likely to perform effectively.

Moving forward, whale shark researchers undertaking telemetry studies should aim to record clear and concise notes on clamp design and attachment position for individual deployments and include this information when publishing tracking datasets. Future studies could also adopt more targeted and methodologically controlled designs, supported by improved sample sizes and planned prior to tagging. For example, externally attached PSATs deployed on blue sharks, *Prionace glauca*, in the northwestern Pacific were evaluated in terms of tag retention and post-release mortality through a structured experimental design [68]. This approach allowed identification of the most effective attachment methods to reduce drag and tag movement, including pre-treating tags with anti-biofouling agents, aligning tags along the anteroposterior axis of the body, ensuring dart penetration of the vertical septum, and fixing tags to the dorsal fin [68]. For whale sharks, future work could similarly refine the attachment methods proposed here using a comparable experimental framework.

## Conclusions

Given that drilling-based attachment is opportunistically rare and logistically challenging for whale sharks, and that dart-based methods with longer tethers – needed for effective satellite relay – often have short retention times and require highly experienced personnel, clamps appear to be the currently recommended option for tagging whale sharks in most field situations when deployed according to best practices. Clamps can provide data of quality and quantity comparable to that obtained via drilling while reducing logistical complexity and enhancing safety for both animals and personnel. They are also likely to minimise potential welfare issues in that they avoid the need to prolong opportunistic capture of individuals and invasively drill into the fin, and they are likely to have a much smaller impact on drag compared to tethered devices, though this has yet to be quantified directly. While clamps were designed to be minimally invasive some still resulted in moderate localised physical fin damage. Our results suggest that more severe injuries were associated with incorrect sizing or placement of clamps, compression due to over-tight clamps, or the use of abrasive hardware. Although some degree of damage from attaching tags is inevitable, it is crucial to minimise this by applying the best available practices. Visible fin damage may negatively influence public perception, especially in ecotourism-linked study areas where research and tourism often overlap [69]. This highlights the importance of developing and continuously evaluating standardised best-practice guidelines on clamp design and deployment, supported by systematic documentation of fin condition and animal behaviour through resighting and photo-ID, although it is acknowledged that this is not always possible. Notably, sharks that were resighted did show full healing from clamp-related injuries; however, longer-term injury and wider welfare impacts remain difficult to assess due to the rarity of repeated observations of the same individuals across multiple years.

In addition to reducing invasiveness, improving clamp design directly increases tag retention times and data quality as was seen in trials of clamp designs D and C (Fig. 5b, c). Recent studies have demonstrated that extended tracking durations from weeks or months to multiple years, enable the detection of broad-scale patterns among multiple individuals such as repeated migrations and long-term site fidelity (e.g., [9]). For whale sharks, extended deployments can allow for the identification of key aggregation sites, cross-boundary movements, and areas of overlap with human activities [11, 36, 41, 42, 70]; insights that are often unattainable with shorter deployments. Similar benefits have been demonstrated in additional shark species and other large marine taxa where extended tracking has provided insight into long-distance movements and habitat connectivity critical for conservation [5, 71]. Moreover, short-term clamp-based archival biologgers optimised for stability and recovery have been used to detect fine-scale behavioural responses to environmental conditions and anthropogenic disturbance [38, 39], with direct consequences for management.

By proposing an improved clamp design in this paper, we aim to facilitate longer and more reliable tag deployments, ultimately enhancing the ecological value of the data collected and allowing for more targeted future studies on attachment performance. The proposed standardised clamp design also serves as a foundation for future refinement, offering a template to improve both deployment longevity and transmission success, as well as the quality of behavioural and environmental data captured. Moving forward, clamp designs should look to integrate advanced materials that can adapt more readily to varied fin compositions, such as carbon composites like Carbon Fibre-Reinforced Polymer. In addition, long-term tags could incorporate mechanisms that enable shedding after a two-year period (or after expected tag battery lifetimes) to ensure no remnants remain on the animal; this could involve coupling links made of steel which can rust over desired timeframes, though testing will be needed. These methodological advances are critical for generating robust, long-term datasets that can directly inform conservation strategies for whale sharks and other threatened species experiencing ongoing population declines, ensuring that future biotelemetry efforts can move toward more efficient, minimally invasive, and ecologically informative tracking technologies.

## Supplementary information

Below is the link to the electronic supplementary material.

Supplementary Material 1

Supplementary Material 2

## Data Availability

Raw information provided by researchers who filled out the questionnaire is not available publicly but has been anonymously summarised throughout the manuscript. The work included no original code.
