# Supplementary material for: Methods for tagging whale sharks: insights into performance and best practices with a focus on clamp attachments
Source: Anim Biotelemetry. 2026 May 5;14(1):22. doi: 10.1186/s40317-026-00462-4 (PMC13263205; doi:10.1186/s40317-026-00462-4)
Supplement: Supplementary file 2 — Supplementary Material 2 [file 40317_2026_462_MOESM2_ESM.pdf]

Supplementary figures

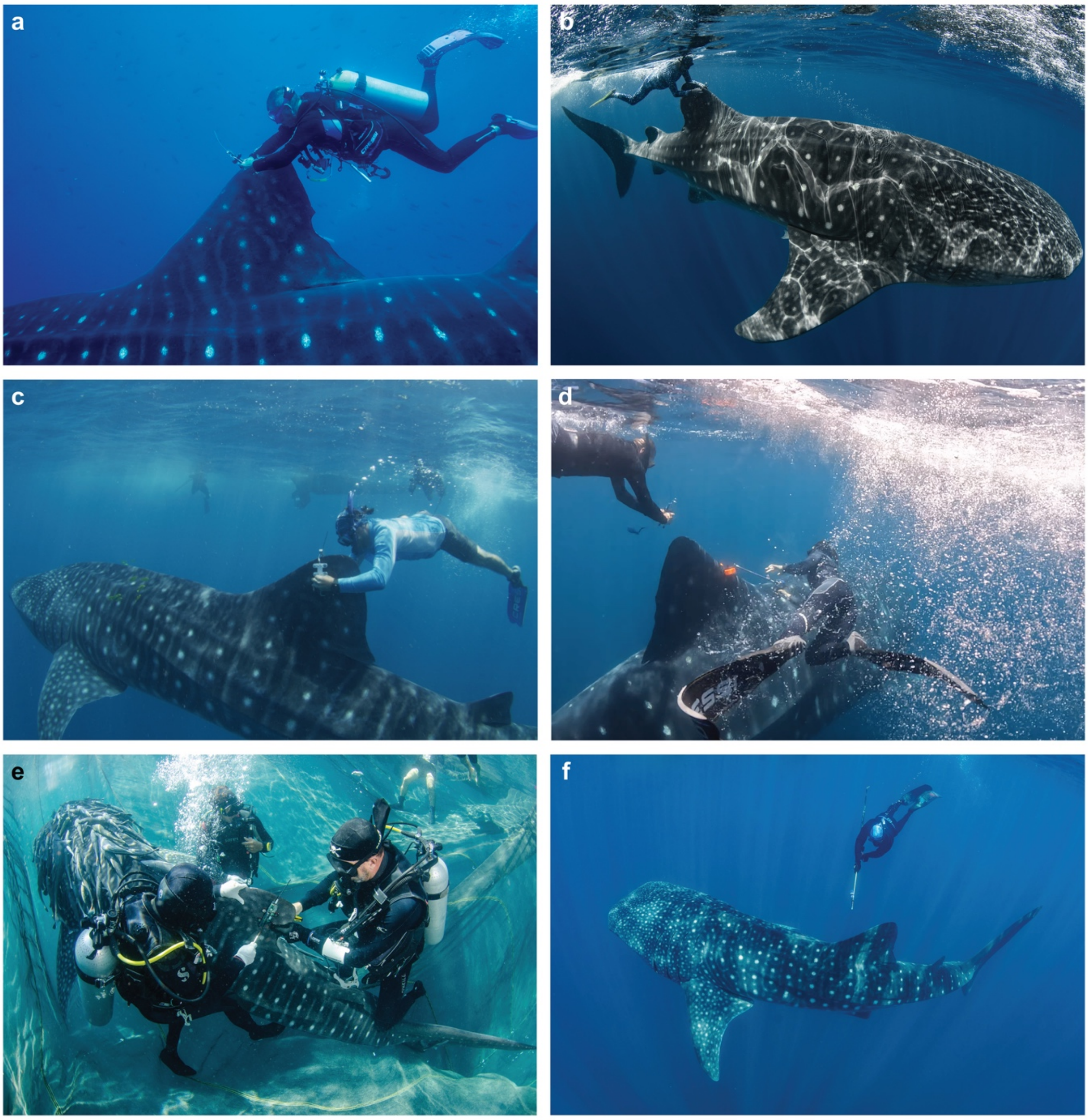

Figure S1 | Examples of long-term (i.e., satellite tag) and short-term (i.e., archival data logger) deployments on whale sharks in **a)** Galápagos, Ecuador (satellite tag deployed via SCUBA, photo by Martin Narvaez), **b)** Yucatán, Mexico (satellite tag deployed via free diving, photo by Rafael de la Parra), **c)** Australia (satellite tag deployed via free diving, photo by Simon Pierce/Biopixel), **d)** La Paz, Mexico (satellite tag and archival data logger synchronously deployed via free diving with use of a deployment aid for the archival tag, photo by Ronan Conlon/Marine Biological Association). **e)** Shows a long-term deployment using drilling methods (satellite tag deployed via SCUBA, photo by Ricard Buxo Kaimana), and **f)** shows a long-term deployment using darting methods (satellite tag deployed via free diving, photo by Simon Pierce).

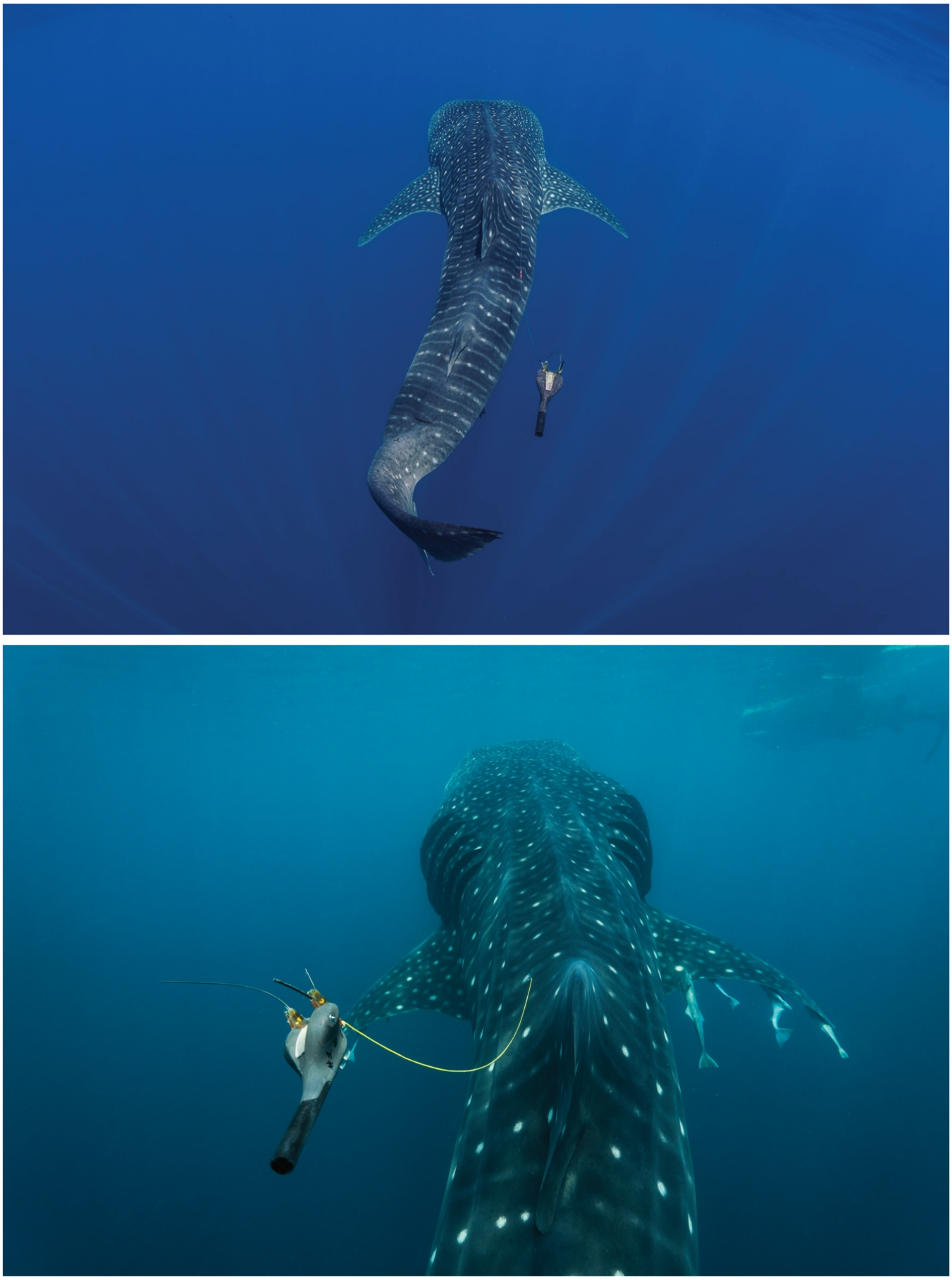

Figure S2 | Examples of Wildlife Computers SPLASH10-F-312 towed tags deployed using a long (> 1 m) tether darted into the muscle in the right (upper panel, photo by Simon Pierce) and left (lower panel, photo by Gonzalo Araujo) flank at the base of the first dorsal fin and incorporating swivels (in the upper panel).

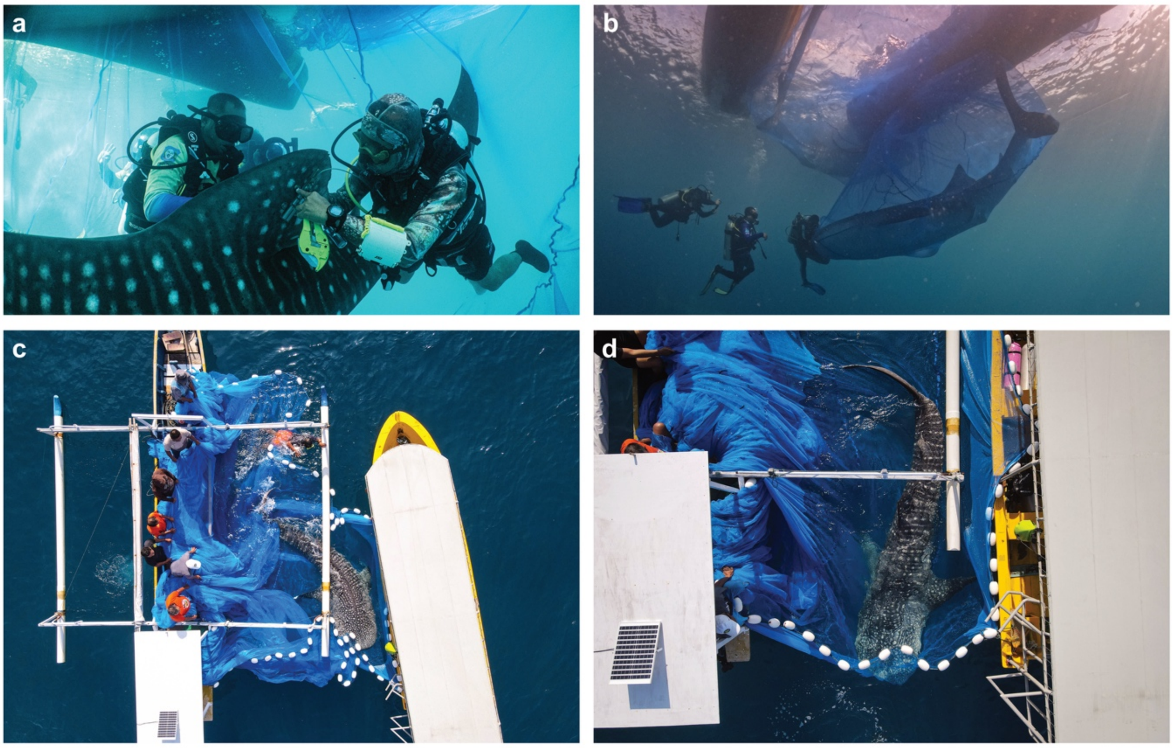

Figure S3 | Examples of drill-based attachment procedure for long-term satellite tags deployed in mini purse seine nets with the help of local fishers in Indonesia. Photos by **a**) Iqubal Herwata, **b**) Abdi Hassan, **c**) and **d**) Tim Monitoring Hui Paus Botubarani

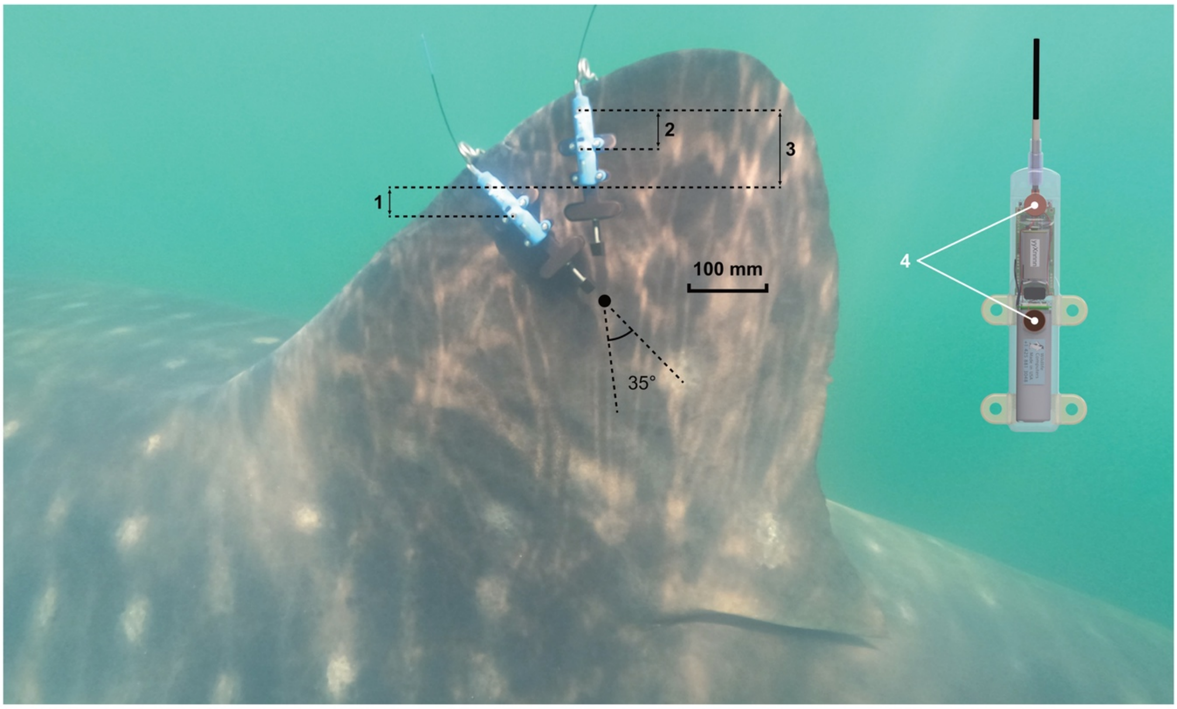

Figure S4 | Demonstration of tag and clamp position optimisation showing the theoretical consequences of repositioning a Wildlife Computers SPOT-420 tag. Position b-1 (left tag, actual position) and position c-2 (right tag, simulated position) are displayed with an approximate angle change of 35-degrees. Here distance 1 measures 35 mm, 2 measures 48 mm, and 3 measures 95 mm, while 4 shows the location of the wet-dry sensors on the SPOT-420. The tag diagram was sourced from Wildlife Computers and the photo is by Alberto García-Baciero. The wet-dry sensor on this device (and similar Wildlife Computers devices) functions by detecting whether the tag is submerged in water or exposed to air, using a conductivity-based system. It consists of two exposed metal contacts that form an electrical circuit (4). When both switches are submerged, the conductivity of the surrounding saltwater allows a small electrical current to flow between the contacts, signalling a "wet" state, and when one of the switches is out of the water, the circuit is broken, and the sensor registers a "dry" state. Therefore, the vertical distance between these two switches determines how easily the sensor can detect whether the tag is in water or exposed to air. The best chance for Argos transmissions (a minimum of three transmissions are needed to derive a reasonably accurate location using the Doppler shift in tag transmission frequency) is for the tag to transmit as soon as the base of the antennae is out of the water.

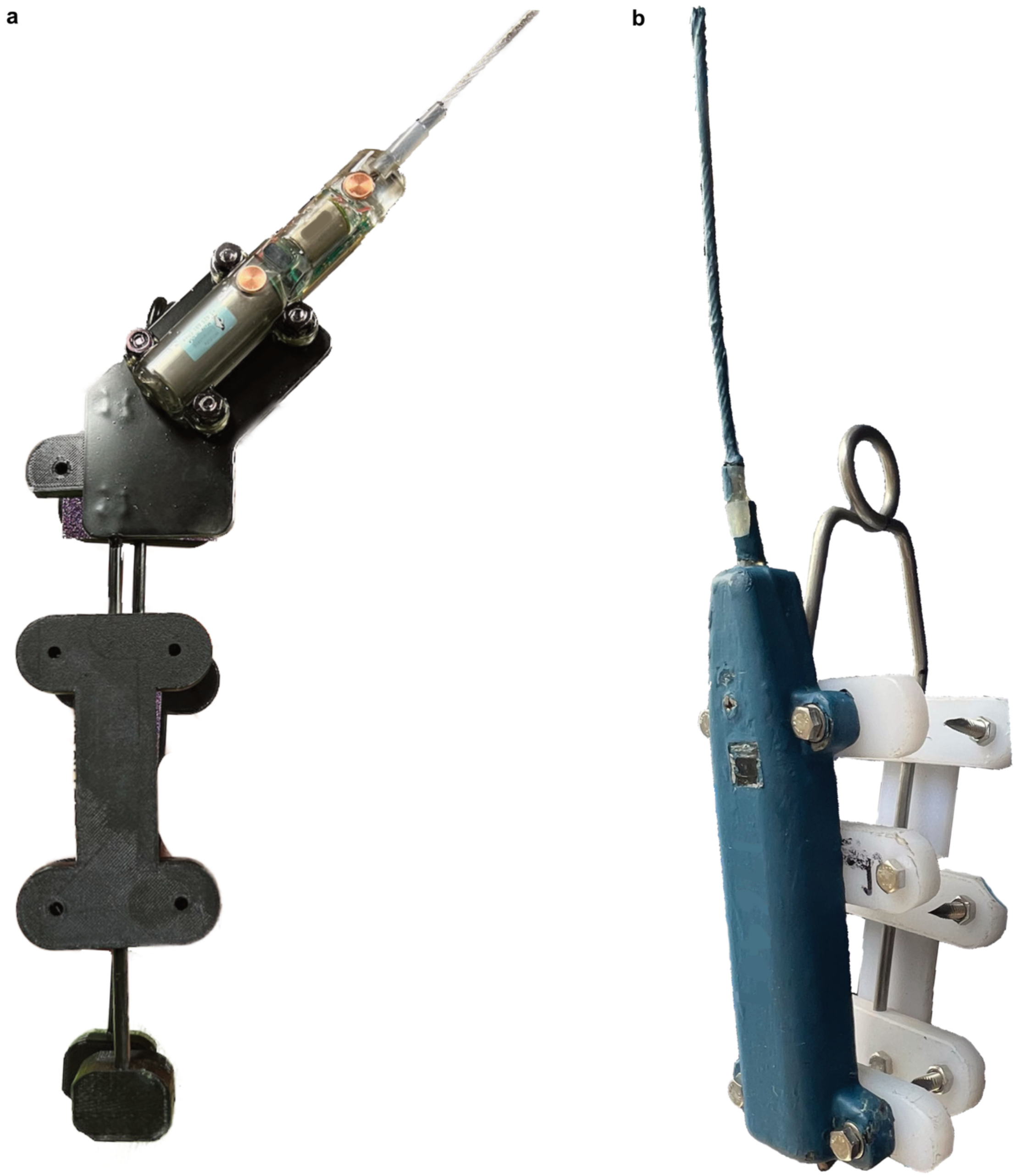

Figure S5 | Examples of designs **a)** B and **b)** D used in the clamp performance analysis.

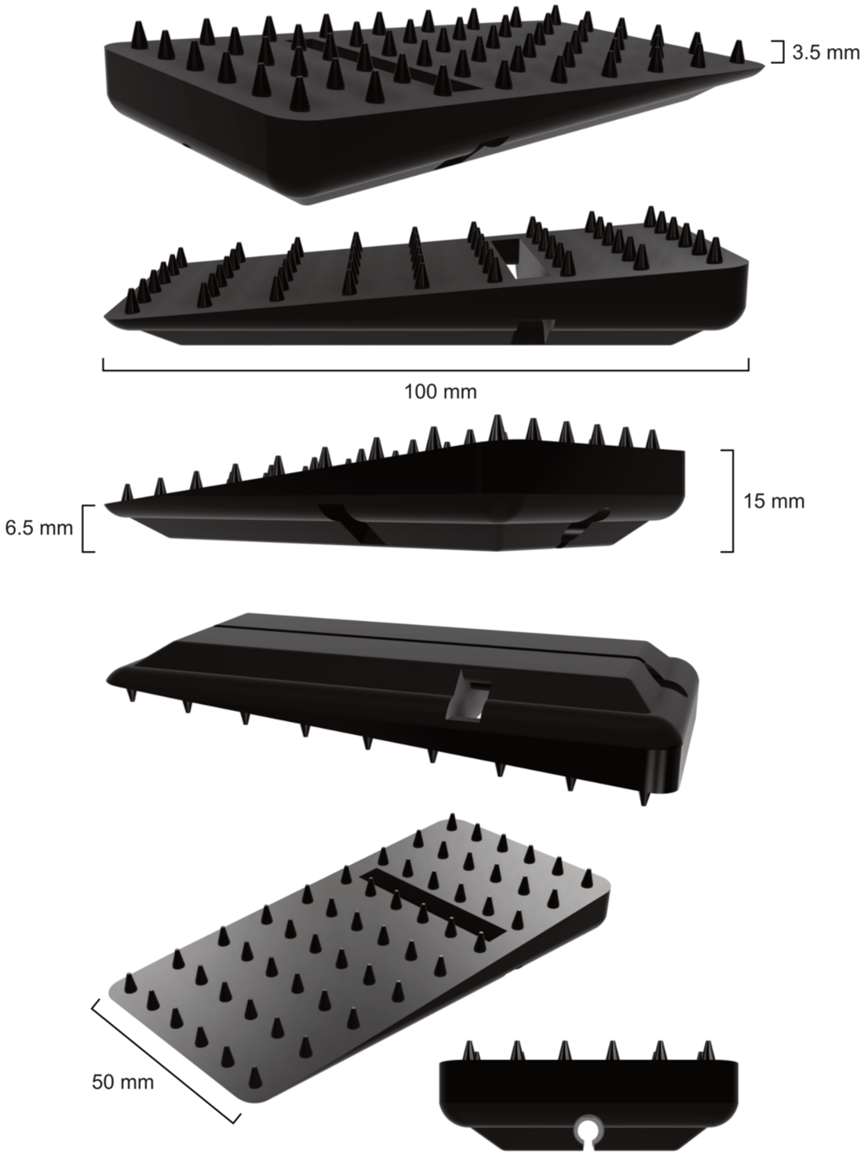

Figure S6 | Schematic illustration of Polylactic Acid (PLA) ‘spike plates’ trialled for short-term clamp deployments with high success rates.

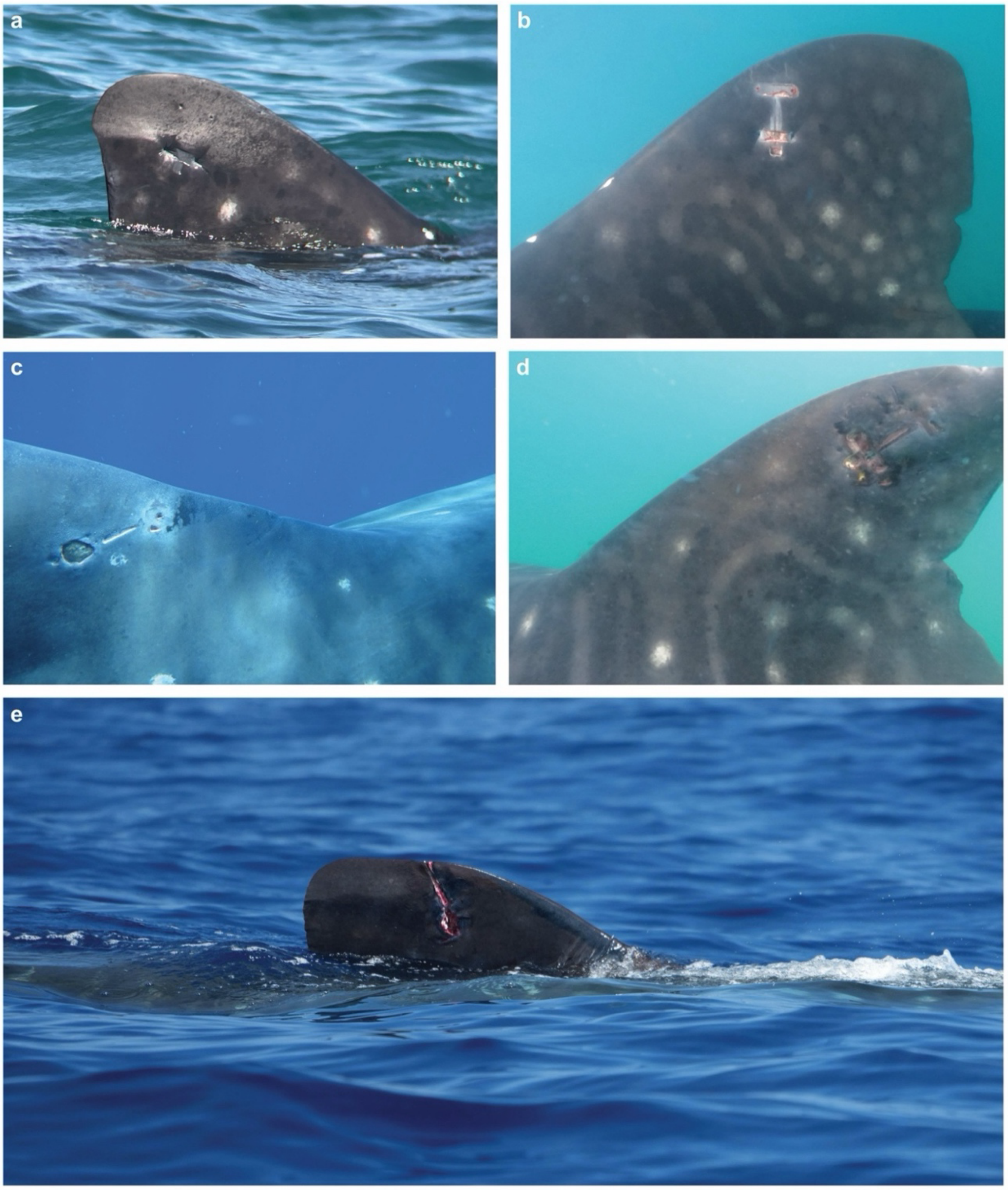

Figure S7 | Examples of potential clamp-related injuries caused by deployment complications. These are only possible to assess in sites where tagged individuals remain for extended periods post-tagging. **a)** shows a non-penetrating compression wound resulting from a tag with excessive tension in the lower section (i.e., < 30 mm at the tail), **b)** shows surface skin irritation (which seems to occur from any foreign object placed on the skin for an extended duration) and a compression wound that has led to deeper layer tissue damage caused by placing a small fitting clamp on an large size individual (> 7 m), **c)** shows tissue damage caused by spike pads and the GTR reactive steel wire, **d)** shows tissue damage caused by a clamp deployed in position c-3 and slipping backward over the top of the fin (when in this position spikes are moved in the flow and can damage the skin), and **e)** shows the upper dorsal fin following clamp removal after ~2 years. Similar injuries show healing within weeks to months (Figure 2). Photos provided by various authors and collaborators.

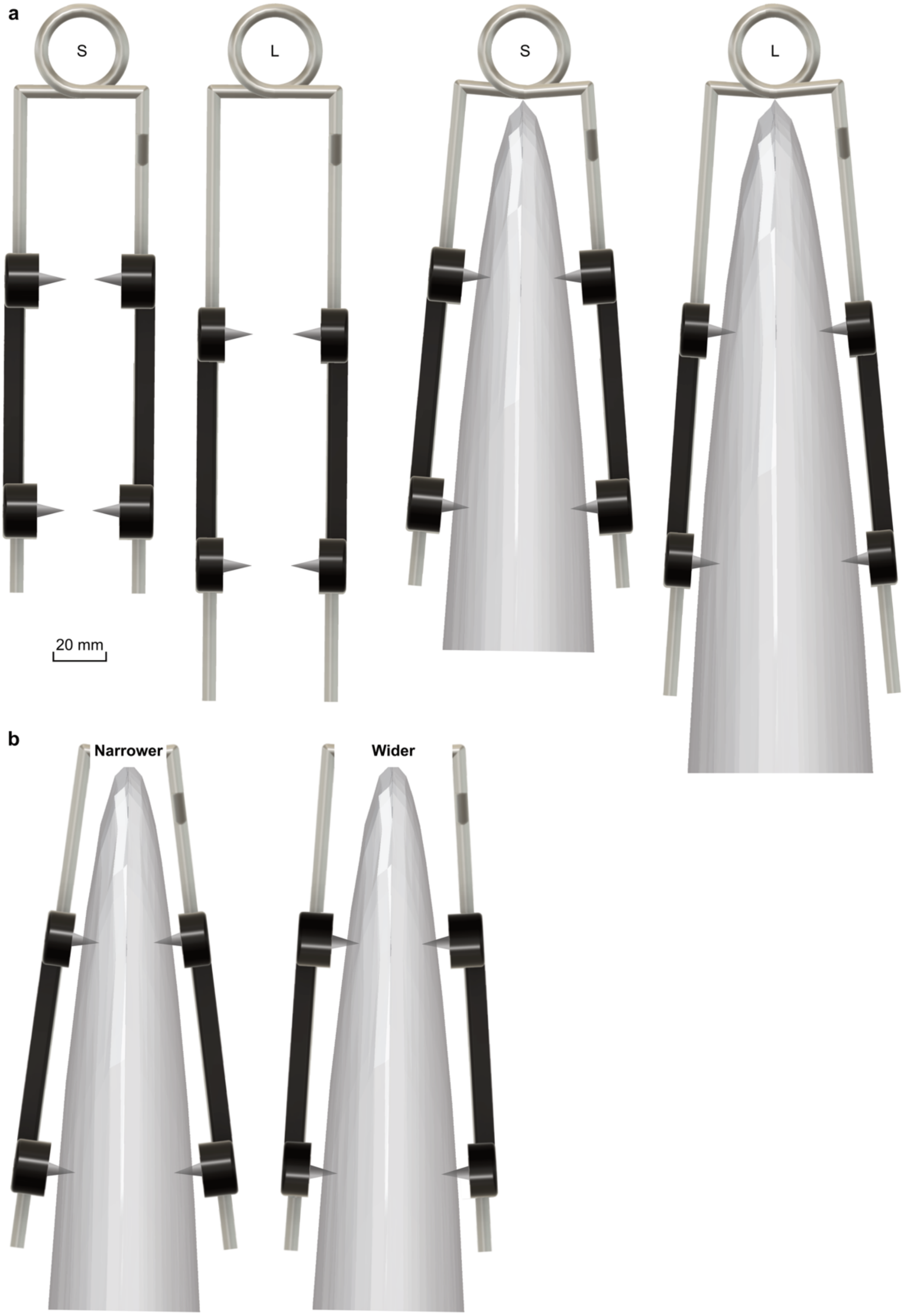

Figure S8 | Visualisations of clamp measurements (provided in Table 1) showing, **a)** small (far left) and large (centre left) suggested designs (40 mm bridge distance) to scale and displayed in an open form on a fin (small, centre right; large, far right). **b)** demonstrates the need to increase the distance between either the top or bottom sections of the attachment pads based on narrower or wider bridge distances to achieve equal contact and tension across the pad. A design that is too narrow without pad thickness adjustments is shown *in-situ* in Figure S8. The fin model was created by DigitalLife3D and sourced from Sketchfab (www.sketchfab.com).

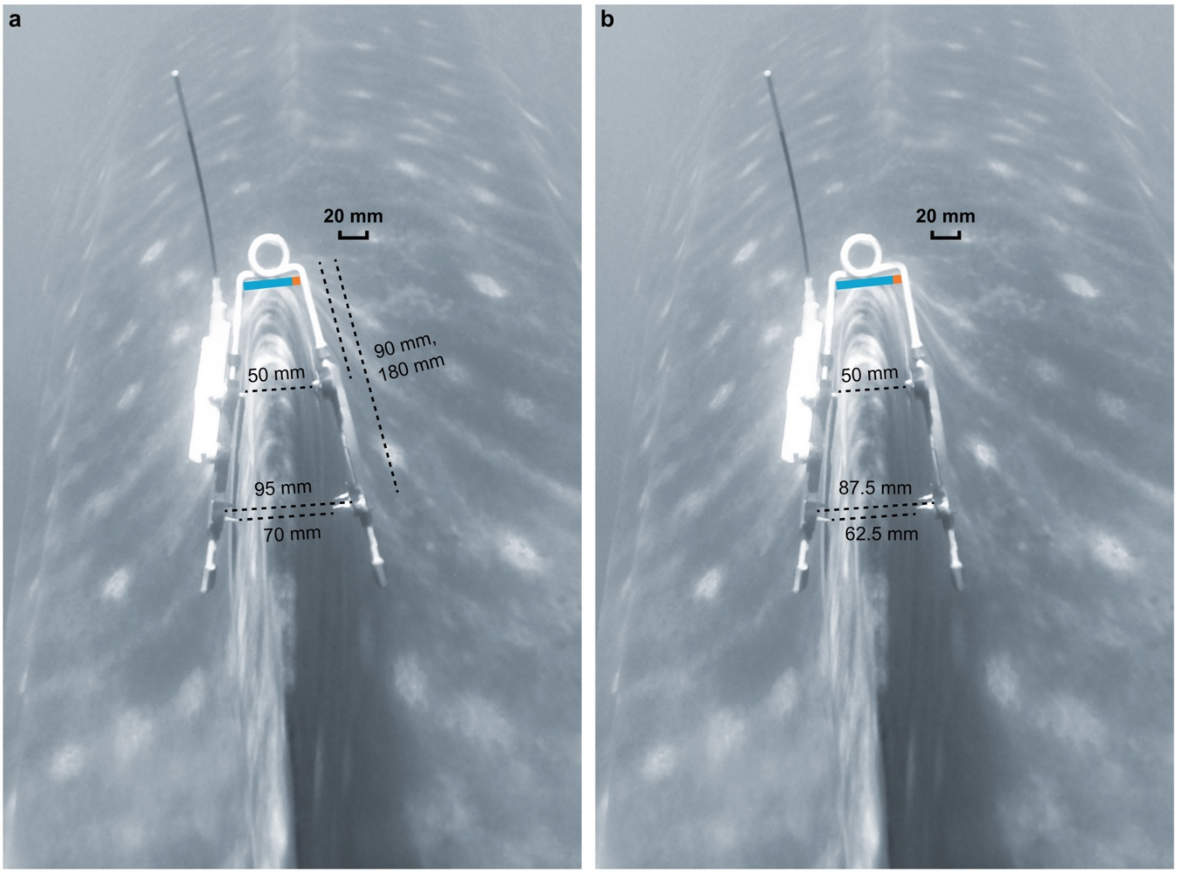

Figure S9 | Demonstrations of tag design optimisation showing the consequences of positioning this tag too low (position b-1) on clamp fit (**a**), and possible solutions (**b**). Because the bridge distance is too narrow for this location on this individual (12 m total length), the lower clamp arms are not able to grip the lower portion of the fin due to the contact of the upper section (this is visualised in Figure S7b). The blue line shows the resting clamp bridge distance, and the orange line shows the additional distance when opened. An adjustment of 5 mm to this distance (from 35 mm to 40 mm, shown on the right panel) adds 7.5 mm to the lower section of the clamp. If also placed higher on the fin where it is thinner (i.e., position b-2) both the upper and lower spikes will contact the fin surface. The adjustments suggested may improve tag retention. Photo by Alberto García-Baciero.

Supplementary tables

Table S1 | List of questions.

Question
Please enter the first year you started tagging whale sharks with satellite tags?
Please enter the country(s) where you have attached satellite tags onto whale sharks?
How many whale sharks have you tagged?
What methods have you used to attach satellite tags onto whale sharks? Tick all that apply.
How have you attached satellite tags onto whale sharks? Tick all that apply, by deployment aid we mean any device which assists in the opening and closing of a clamp or firing of a dart.
If you have tagged using drilling methods, how did you find this?
For tagging using clamping methods, how did you find this?
What year did you start using clamps specifically?
How many field expeditions have you been on/conducted where clamps were used to tag whale sharks?
What type of satellite tags have you deployed using clamps? Tick all that apply.
How many individuals have you tagged using long-term (e.g. spot, splash, psat) clamps? If you know the exact number please state in other.
Overall how many long-term (e.g. spot, splash, psat) tags have you attached to whale sharks?
Have you double tagged using clamps (i.e. spot and psat together)?
How many individuals have you tagged using short-term (e.g. accelerometer, video) clamps? If you know the exact number please state in other.
Overall how many individuals that you tagged using long-term (e.g. spot, splash, psat) clamps transmitted during the first 7 days? If you know the exact number please state in other.
Overall how many individuals that you tagged using long-term (e.g. spot, splash, psat) clamps transmitted after the first 7 days? If you know the exact number please state in other.
How many individuals have you seen days after clamping in order to assess the attachment and impacts?
How many individuals have you seen weeks after clamping in order to assess the attachment and impacts?
How many individuals have you seen months after clamping in order to assess the attachment and impacts?
What size are the individuals that you have tagged with clamps? Tick all that apply.
Have you ever worked with external providers to purchase 'off the shelf' components? e.g. CATS, please provide more details in 'Other' if happy to share.
Which 'off the shelf' components did you purchase? Tick all that apply.
Did you deploy 'off the shelf' components?
If 'Yes', which 'off the shelf' components did you deploy/ use to deploy unaltered? Tick all that apply.
If you altered 'off the shelf' components prior to deployment, how did you alter them? Please provide more details in 'Other' if needed.
Have you designed and developed your own components? If so, please select.
How many iterations of long-term (e.g. spot, splash, psat) clamp designs have you deployed with notable alterations between each?
Do you have plans to change your clamp design in future to improve on current methods?
What is the most important factor you consider when developing long-term clamps?
Have you encountered any of the following known setbacks in the field?
Attachment angle of the tag: Which of the above angles have you used for positioning the tag? Tick all that apply.
Attachment angle of the tag: Which option worked the best for you in terms of data quality?
Attachment angle of the tag: Please provide more information. For example, the average number of daily satellite locations for the best performing option.<br>Did you try any particular option that didn't work well?

Attachment angle of the clamp: Which of the above angles have you used for positioning the clamp? Tick all that apply.
Attachment angle of the clamp: Which option worked the best for you in terms of tag retention, impact on the animal, and data quality?
Attachment angle of the clamp: Please provide more information. For example, the average number of days the clamp stayed on for the best performing option.
Did you try any particular option that didn't work well? Did some options lead to the clamp changing position over time or falling off prematurely?
Attachment of the clamp: Please outline roughly how many mm's are designed to be between the clamp ring and the leading edge of the fin.
Attachment pads: Please outline methods you have used to attach the tag onto the clamp and to secure the clamp onto the fin. This can potentially affect tag retention, and impact on the animal.
Attachment pads: Please select any attachment pads you have used. Tick all that apply.
Attachment pads: Which option worked the best for you in terms of tag retention and impact on the animal?
Attachment pads: Please provide more information. For example, did you notice any slipping of the plate position with time? Did they cause any damage to the fin?
If you used spikes, what were they made of and what length were they?
Attachment pads: Did you design your own attachment pads?
Attachment pads: If 'Yes', please provide more information on the design (i.e. material used, general shape, length of spikes).
Attachment pads: If multiple pads were used, please outline how many, where these were positioned, and whether these were secured in place on the spring steel wire.
Steel wire measurements: Based on the above diagram please detail which clamp measurements you have deployed. If you bend the wire please detail how far down the arm you bend f and the angle g, in addition d represents the bottom of the arms and c mid way up. Please also note the size of the animals a design was intended for. If you have deployed more than one design iteration please number each.<br>For example, two iterations could read as follows:<br>1) size: 5m, a: 25mm, b: 40mm, c: 35mm, d: 35mm, e: 190mm, f: 100mm, g: 180deg, h: 90 deg.<br>2) size: 10m, a: 35mm, b: 45mm, c: 45mm, d: 45mm, e: 220mm, f: 50mm, g: 190deg, h: 85 deg.
Steel wire measurements: Which measurements worked the best for you in terms of tag retention and impact on the animal? Please include size of target animal. Please copy best from answer above.
Steel wire measurements: Please provide more information. For example, did you notice that certain measurements caused impacts to the animal by being too tight? Were any that you trialed too loose?
Steel wire measurements: How many turns in the steel have you trialed? Tick all the apply.
Steel wire measurements: On a scale of 1 to 10 how tight were the clamps you used? How difficult were they to open by hand?
Steel wire measurements: Did you alter clamp tension across deployments? If so, please provide details.
How many days did/has your most successful clamp design remain/ed attached to a whale shark?
Please use this section to provide any more information you feel is relevant to tag retention, impact on the animal and data quality. Or anything else you would like to share regarding long-term clamps.
If you would like to share any insight on short-term clamping methods, please do so here.
Have you used Galvanic Timed Releases (GTRs)? If so please provide details.
If you have drilled into the fin please describe the technique.
If you have drilled into the fin please describe any setbacks/failures. i.e. damage to the animal or difficulties deploying.
What materials have you used for the bolts when drilling into the fin?
If you have drilled into the fin please describe any successes. i.e. long retention or high data quality.
If you have fired tethered darts into the fin or flank please describe the technique.
What length of tether have you trialled.
If you have fired tethered darts into the fin or flank in please describe any successes. i.e. long retention or high data quality.
If you have fired tethered darts into the fin or flank please describe any setbacks/failures.

Table S2 | Dart-based method insights pooled from the survey responses and lightly edited for grammar and clarity.

How?	Length	Comments
Sling with the standard dart	1.2 m	Long retention<br>Tourists removing tags
Titanium dart and pole spear (Wildlife Computers)	1 to 3 m	Lots of successes but lots of failures: 6-month full deployment with PSATs.<br>Adjusting power for skin thickness.
Spear guns and Hawaiian sling hand spears, usually with two elastics to provide sufficient force.	1.2 m for SPOT, < 15 cm for PSAT.	Best results below the base of 1st dorsal.<br>Premature release due to crush depth violations.
Pole spear and intradermal dart to anchor the tag.	Short for PSAT, much longer for SPLASH and SPOT tags.	We have better success rates with tags that have less tether length. The limiting factor for some of our tags is the deep diving behaviour.<br>The longer the tether the lower the retention time. Likely due to other animals investigating and pulling the tags out of the animals and entanglement, since whale sharks grasp their backs on objects. The pole spear shot and the quality of anchor insertion are limiting factors for shorter tethered tags.
Pole spear with titanium dart	15 cm for PSAT<br>1.5 m towed SPOT	We had several PSATs make it a year, but the towable SPOT only lasts 2-3 months.<br>Tether material is critical as the skin interface can cut through the tether material.
Into flank	1.5 m	Retention times are usually only a few weeks or months.<br>Our observations of other research groups show that if the dart was fired deep into the muscle layer, then longer retention times were possible. However, these caused considerable damage to the flank of the shark due to the drag of the tag and tether.<br>We abandoned this approach fairly quickly due to the short retention times.
Pneumatic spearguns	15 cm for PSAT and 1 m for other towed tags	Retention barely exceeds 5 months.<br>Biofouling and algae contribute to the shedding.
Titanium dart anchors deployed by Hawaiian sling spear into the anterior base of the dorsal fin.	15 cm to 2 m	Our PSAT tags were programmed to pop off at either 6 or 9 months and we had several complete these programmed deployments. Longer-tethered SPOT and SPLASH tags have typically lasted for a few months
Hand spear and pneumatic spear guns	50 cm to 180 cm (hand and pneumatic, respectively)	Retentions ranging from one week to one year.<br>Shooting high-power pneumatic guns can damage the tags if the tether is shorter than the shooting distance.<br>Penetration of large animals' skin can be difficult and is location-dependent (base of the first dorsal).
Adapted pole spear (Hawaiian sling) with a special adaptor.	10 cm to 1.5 m	The dart of the tag was inserted in the adaptor beforehand and secured with an elastic band. Tags were connected to a small titanium dart (WC) via a ~150 cm tether of 240 kg Dyneema braided line. Then when the right shark passes, we use the elastic to insert the tag under the skin and aim for the area

		under the dorsal fin on the left flank, so that the tag floated approximately above the first dorsal fin.<br>Long retention, detailed location with smaller error radii.<br>Big issues with the long tethers: risk of whale shark & swimmer entanglement, visibility making them removable, negative comments from the community (both operators and tourists, rare misses of tag insertion, and no retrieval possible, leading to pollution.
Hawaiian-sling pole-spears for up to ~8 m sharks, pneumatic spearguns for larger animals.	80 cm to 1.5 m	Aiming point is typically in the depression under the uppermost dorsal ridge, with the dart inserted at right angles (i.e., perpendicular) in relation to the shark's body.<br>Outside the Galápagos, where predation on tags was an issue, we typically had reasonable retention comparable to the mean durations from fin-clamps. Because many detached tags washed ashore, we were able to identify weak points.<br>We eliminated swivels, crimps, and wire traces, settling on a length of Dyneema spearfishing line tied directly to the dart at one end and the tag at the other.
Hawaiian sling for sharks <6 m, and double rubber spear gun for >6 m with titanium dart.	1 to 2 m	Always use the ~6 cm titanium darts (WC) for towed tags. For smaller tags used the small titanium darts.<br>Retention doubled with longer applicators: the standard WC ones are too short. So we had special, longer ones made, and it more than doubled the retention time of towed tags.<br>The shark flexing/moving more than 20 degrees during firing affects penetration of the applicator and dart, thus affecting retention. Patience is key to ensure a parallel side of the shark to the shooter.
Pole spear with a hardened steel insertion tip (WC). The insertion tip has a hardened "stopper" on it to prevent the dart tip from going more than 10 cm into the dorsum of the whale shark.	15 cm for PSAT, 1.50 m for other towed tags.	For the 150 cm tether, we inserted a high-quality Japanese ball-bearing fishing swivel in the middle of the tether to prevent coiling of the tether - which has worked very well for us on manta rays and also worked well on whale sharks.<br>With the PSATs we deployed, they generally had excellent retention.<br>With the SPLASH10F, we were very happy with how the tag "flew" behind and above the shark on a 150 cm tether. Unfortunately, one of the two tags we deployed on whale sharks never transmitted.<br>We've found that with tethers longer than 75cm (stainless steel), there is a strong likelihood that the tether will "supercoil" from the animal's movements. We have countered this by always inserting a high-quality Japanese fishing swivel (with ball bearings) into the middle of the tether, which seems to prevent supercoiling of the tether

Spear pole with custom-made tip applicator	30 cm PSATs to 1.5m (SPOT, SPLASH tags)	We had mixed successes. Some PSAT tags remained attached for the full duration of their programmed deployment (up to 360 days), while others failed to transmit entirely (assumed tag malfunction), and several tags released prematurely (mostly due to max depth reached). Tethered Spot tags (model 253) similarly showed variable performance, with retention times of up to five months. However, while active, they provided high-quality location data. As we received no further transmissions beyond five months, we suspect tag loss due to predation or other external factors that may have caused damage or tag loss.<br>Tag failures (PSAT/SPLASH10-F never reported back); Spot-253: pinging but not transmitting locations despite good voltage, but low power (low dB), indicating possible damage to antenna, potentially predation<br>Premature release: PSAT tags due to pressure release (i.e., too deep) and pin still intact; SPOT tag: tag came off, washed ashore
Hand spear or double rubber speargun	2 m	Limited success
Hand spear with an elastic to place the dart into the flank below the 1st dorsal. Also a spear gun for larger sharks.	10 cm for PSAT 1.8 m for SPOTs	SPOT tags: mean of 5 locations per day, 2.6 locations with good ARGOS quality. Acoustic tags with short tethers up to 5-year retention (exception, mean was maybe ~330 days).<br>I saw one of the SPOTs with a long tether once where a sinker of a fishing line setup got entangled, causing the tag to be below the shark (we cut it off and redeployed on another shark later).

Table S3 | Number of individuals transmitting in response to the questions 'How many individuals have you tagged using long-term (e.g., SPOT, SPLASH, PSAT) clamps? If you know the exact number please state in other' (column 1), 'Overall how many individuals that you tagged using long-term (e.g., SPOT, SPLASH, PSAT) clamps transmitted during the first 7 days? If you know the exact number please state in other' (column 2), and 'Overall how many individuals that you tagged using long-term (e.g., SPOT, SPLASH, PSAT) clamps transmitted after the first 7 days? If you know the exact number, please state in other' (column 3).

N tagged using long-term clamps?	N transmitted during the first 7 days?	N transmitted after the first 7 days?
75	51	45
1-5	5	2
11-20	11-20	11-20
12	12	12
6-10	6-10	1-5
21+	12	8
11-20	6-10	1-5
1-5	1-5	1-5
21+	21+	6-10
31	21+	21+
66	66	64
24	24	22

Table S4 | Number of individuals sighted in response to the questions 'How many individuals have you seen [days (column 1)], [weeks (column 2)] or [months (column 3)] after clamping in order to assess the attachment and impacts?'.

Days	Weeks	Months
4	0	0
2	0	0
8	1	0
0	0	1
5	0	0
0		1
10-15	0	0
5+2	2	0
8		2
5	2	0
0	0	0
~20	~3	0
5	0	0
>40	<5	3
5-10	0	0
~10	~15	~10
3	0	2
15	15	1

Table S5 | Total track duration (days), number of days with locations recorded, and mean transmissions per day are shown for drill-based tags and four clamp designs (A - D). Values are presented as mean, standard deviation (SD), and median. Sample sizes are n = 44 for drilled tags, n = 8 for Design A, n = 14 for Design B, n = 12 for Design C, and n = 18 for Design D. When calculating transmissions per day (in the first 45 days) sample size fell to n = 7 and n = 9 for Designs C and D, respectively.

	**Duration (days)**		
**Design**	**Mean**	**SD**	**Median**
Drilled	514.61	240.33	498.00
A	233.63	122.38	257.00
B	14.64	21.81	2.00
C	399.17	48.58	423.50
D	152.83	234.81	71.00
	**Days with locations (days)**		
	**Mean**	**SD**	**Median**
Drilled	149.59	80.10	147.50
A	91.88	57.23	105.50
B	10.21	16.89	2.00
C	184.17	52.23	166.50
D	42.22	50.84	26.50
	**Transmissions (per day)**		
	**Mean**	**SD**	**Median**
Drilled	3.09	1.96	2.50
A	13.10	8.74	11.00
C	13.23	4.90	12.90
D	6.86	4.89	4.63

Additional acknowledgements

C.B. was supported by an Australian Government Research Training Program (RTP) Scholarship doi.org/10.82133/C42F-K220.

A.H., E.S., A.B.S. & M.V.E. thank MAC3 Impact Philanthropies for support of their whale shark tagging work, and the Cendrawasih Bay National Park Authority (BBTNTC) and Kaimana regional government for permitting our tagging work in West Papua.

E.H, J.H., J.S., R.C. and J.L. extend their sincere gratitude to the numerous individuals who contributed to our whale shark tagging efforts over the past few years. We thank Carlton Ward Jr./Wildpath for providing an image utilized in this manuscript, which was captured during recent research trips. J.H. acknowledges the University of Southern Mississippi Institutional Animal Care and Use Committee #9031204, 11092203 and 18010501. This work was financially supported by the Bureau of Ocean Energy Management under award number 140M0123F0005.

S.J.P. and C.A.R. thank Waterlust, the Shark Foundation, Aqua-Firma, Nacora and other Marine Megafauna Foundation (MMF) funders for supporting the global whale shark research program at MMF.

P.A., J.F. and B.C.L.M. were funded by the projects IslandShark (FCT-PTDC/BIA-BMA/32204/2017; Oceanário de Lisboa and the Shark Conservation Fund), EcoDivePWN (proWIN proNATURE Foundation) and AEROS-Az (ACORES-01-0145-FEDER-000131), by the European Commission through the projects MEESO (H2020-LC-BG-03-2018–817669), Mission Atlantic (H2020-LC-BG-08-2018-862428), NAUTILOS (101000825 H2020-BG-2018-2020/H2020-BG-2020-1) and OceanICU (101083922). Also, from National funds through FCT, I.P., under the Strategic Program to Okeanos R&D Centre (UIDB/05634/2023 and UIDP/05634/2023) and Regional funds from the Government of the Azores (M1.1.A/FUNC.UI&D/003/2021-2024 and M1.1.A/REEQ.CIENTÍ FICO UI&D/2021/010).

S.S.R. was supported by the Fundação para a Ciência e Tecnologia (FCT) through the PhD Research Grant 2022.11531.BD. and NGANDU (AGA-KHAN/541746579/2019).

I.B.M. acknowledges support through an Australian Government Research Training Program Scholarship.
